# Land Use and Land Cover Change Dynamics across the Brazilian Amazon: Insights from Extensive Time-Series Analysis of Remote Sensing Data

**DOI:** 10.1371/journal.pone.0104144

**Published:** 2014-08-06

**Authors:** João M. B. Carreiras, Joshua Jones, Richard M. Lucas, Cristina Gabriel

**Affiliations:** 1 Tropical Research Institute (IICT), Lisboa, Portugal; 2 National Centre for Earth Observation (NCEO), Centre for Terrestrial Carbon Dynamics (CTCD), University of Sheffield, Sheffield, South Yorkshire, United Kingdom; 3 Department of Geography and Earth Sciences, Aberystwyth University, Aberystwyth, Ceredigion, United Kingdom; 4 Centre for Ecosystem Science, School of Biological, Earth and Environmental Sciences, University of New South Wales, Sydney, New South Wales, Australia; Cirad, France

## Abstract

Throughout the Amazon region, the age of forests regenerating on previously deforested land is determined, in part, by the periods of active land use prior to abandonment and the frequency of reclearance of regrowth, both of which can be quantified by comparing time-series of Landsat sensor data. Using these time-series of near annual data from 1973–2011 for an area north of Manaus (in Amazonas state), from 1984–2010 for south of Santarém (Pará state) and 1984–2011 near Machadinho d’Oeste (Rondônia state), the changes in the area of primary forest, non-forest and secondary forest were documented from which the age of regenerating forests, periods of active land use and the frequency of forest reclearance were derived. At Manaus, and at the end of the time-series, over 50% of regenerating forests were older than 16 years, whilst at Santarém and Machadinho d’Oeste, 57% and 41% of forests respectively were aged 6–15 years, with the remainder being mostly younger forests. These differences were attributed to the time since deforestation commenced but also the greater frequencies of reclearance of forests at the latter two sites with short periods of use in the intervening periods. The majority of clearance for agriculture was also found outside of protected areas. The study suggested that a) the history of clearance and land use should be taken into account when protecting deforested land for the purpose of restoring both tree species diversity and biomass through natural regeneration and b) a greater proportion of the forested landscape should be placed under protection, including areas of regrowth.

## Introduction

Humans are increasingly changing the state and dynamics of the Earth system, affecting processes within and between the biosphere, hydrosphere and atmosphere. Climate change is a recognized consequence of such changes and would be occurring at a faster rate if several ecosystem services at the global scale were absent [1]. For example, less than half of the total amount of carbon dioxide (CO_2_) released to the atmosphere each year remains there because of removal by terrestrial and ocean carbon (C) sinks [2]. Losses of biodiversity from terrestrial ecosystems have also been substantial.

The role of terrestrial ecosystems in the global C cycle had been widely recognized in the literature (e.g., [3]), with particular recognition given to the pan-tropical belt [4], [5]. In the past two decades, two main international conventions have sought to establish mechanisms aimed at stabilizing greenhouse gases (GHG) concentrations in the atmosphere (the United Nations Framework Convention on Climate Change; UNFCCC) and protecting biological diversity (Convention on Biological Diversity; CBD). Specifically, the alarmingly high rate of tropical land use and land cover change (LULCC) [6] and resulting biodiversity loss with further severe consequences for ecosystem function and structure [7] has driven the UNFCCC to establish several investment mechanisms and market based C transactions. These are related to the enhancement of forest C stocks and the decrease of deforestation and forest degradation, while promoting sustainable development in developing countries (UNFCCC Non-Annex I countries). Such mechanisms include the UNFCCC Clean Development Mechanism (CDM) initiative and the post-Kyoto Protocol Reduced Emissions from Deforestation and Degradation (REDD+) program (e.g., [8]). These efforts are proving successful in many regions leading to greater conservation of the intact forests of the Neotropical, Afrotropical, Australasia and Indo-Malay biogeographical regions [9] and the carbon and biodiversity they contain. However, many of the deforested and degraded lands of the tropics are also capable of supporting forests and hence, in addition, there is potential to restore some of the ecosystem values (e.g., carbon amounts and biodiversity) lost through previous disturbances [10], [11]. This capacity depends in part, however, on the history of forest clearance and land use that has occurred and hence a clearer understanding of the potential of deforested areas to recover ecosystems is needed. This study addresses these issues by focusing on sites in the Brazilian Legal Amazon (BLA) that have experienced deforestation and variable histories of subsequent land use.

The BLA covers an area of approximately 5,000,000 km^2^ and consists primarily of primary tropical forest (rain and seasonal forest) [12]. Brazil’s National Institute for Space Research (Instituto Nacional de Pesquisas Espaciais, INPE) has, since 1988, undertaken annual mapping of deforestation within the BLA using remote sensing data [13], which has been highly variable across the region. For example, an average deforestation rate of 21,050 km^2^ yr^−1^ was reported between 1977 and 1988, which decreased subsequently to 11,030 km^2^ yr^−1^ up to 1991; from 1991 to 2004 the deforestation rate increased to 27,772 km^2^ yr^−1^ (reaching a record high of 29,059 km^2^ yr^−1^ from 1994 to 1995) and then decreased to a record low of 4,571 km^2^ yr^−1^ in 2012 [13]. Currently, much of the deforested area is under agricultural use (following disturbance mainly with slash-and-burn practices) but in many areas, the land has been abandoned and frequently supports forests at different stages of regeneration [14].

Several estimates of the deforested land occupied with regeneration have been generated, mainly from interpretation of remote sensing data, but also using transition modeling. Data from the National Oceanic and Atmospheric Administration (NOAA) Advanced Very High Resolution Radiometer (AVHRR) from 1988–1991 were used by Stone et al., 1994 [15] to generate a land cover map of South America, and subsequently Schroeder and Winjum, 1995 [16] used this dataset to estimate the extent of forest regeneration in the BLA at ∼151×10^3^ km^2^. Fearnside, 1996 [17], using a matrix of annual transition probabilities, estimated that approximately 48% (∼195×10^3^ km^2^) of the deforested landscape in 1990 supported forest regeneration. Lucas et al., 2000 [18] using NOAA AVHRRR data estimated that ∼158×10^3^ km^2^ supported some type of forest regeneration in the period 1991–1994. Cardille and Foley, 2003 [19] used a matrix of annual transition probabilities to estimate that 36% (∼91×10^3^ km^2^) of the area deforested between 1980 and 1995 in the entire Amazon river drainage basin was in some stage of secondary succession forest. Carreiras et al., 2006 [20] exploited a time-series of 12 monthly composite images of the year 2000, derived from the *Satellite Pour l’Observation de la Terre* (SPOT-4) VEGETATION sensor and estimated that, in 2000, ∼140×10^3^ km^2^ of land was occupied by regenerating forests. Neeff et al., 2006 [21] used large-area land cover maps derived from remote sensing datasets to generate estimates of regrowth extent within the BLA and concluded that regrowth increased from ∼29×10^3^ km^2^ in 1978 to ∼161×10^3^ km^2^ in 2002. Whilst providing information on the extent of regenerating forests at certain points in time, the dynamics of regenerating forests and the land on which they occupy has only been attempted using remote sensing by a few studies (e.g., [22]). However, such information is needed across large areas as regenerating forests may represent one of the prime mechanisms by which biomass, biological diversity and ecosystem services can be restored [23]–[25].

The aim of this study was to establish the extent to which the age class distribution of regenerating forests was determined by the history of deforestation and subsequent use and management of the land, thereby giving insight into the potential for forest restoration and conservation into the future. This was achieved by comparing Landsat sensor data classified into the broad amalgamated categories of mature forest, non-forest (i.e., agriculture, including stock pasture) and secondary forest over decadal periods and time-separated by a maximum of four years. Three study areas were chosen to perform the analysis: Manaus (Amazonas state), Santarém (Pará state) and Machadinho d’Oeste (Rondônia state). Several measures were used to compare the three areas since the inception of widespread agricultural practices in the region (1970s for Manaus and Santarém and 1980s for Machadinho d’Oeste) up to the present: Deforestation (conversion from mature forest to non-forest or from secondary forest to non-forest) and regrowth rates (conversion from non-forest to regeneration), the age of secondary forest, the period of active land use prior to abandonment to regeneration, and the frequency of clearance. The study builds on that of Prates-Clarke et al., 2009 [38] in that the time-series was extended from 2003 to 2011 for Manaus and Santarém and a new time-series was generated for Machadinho d’Oeste.

## Study Areas

**Figure 1:**
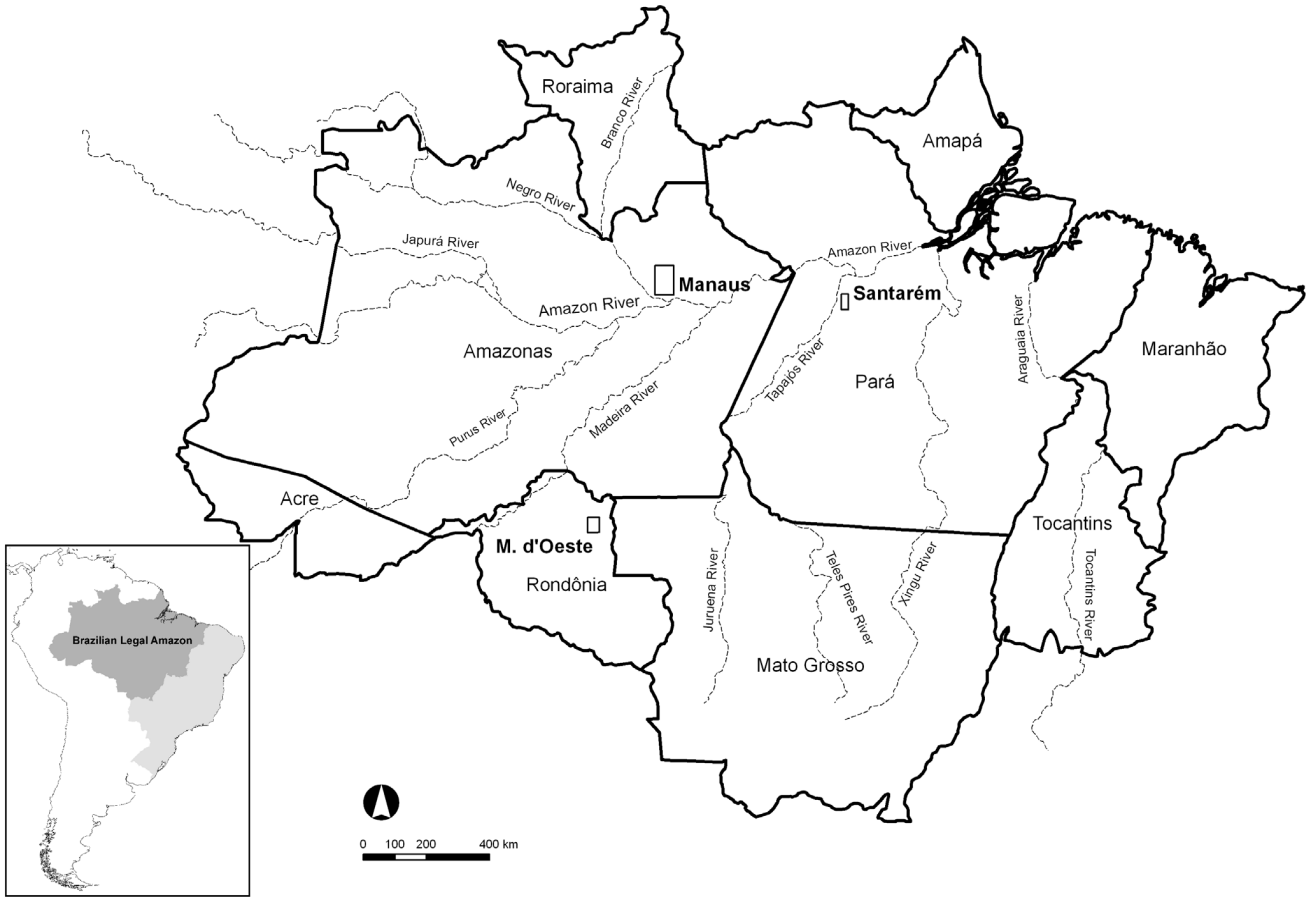
Location of the three study sites. Location of the three study sites: Manaus, Santarém and Machadinho d’Oeste (M. d’Oeste), and the insertion (bottom left) of the Brazilian Legal Amazon (dark grey) in Brazil (light grey) and South American continent. Also shown are the major rivers included in the Amazon basin.

For all three sites (Manaus, Santarém and Machadinho d’Oeste; Figure 1), the natural vegetation prior to disturbance was primary (undisturbed) *terra firme* (i.e., non-flooded) forest. In each case, the canopy heights averaged between 25 and 35 m, with emergent trees exceeding 50 m at the more productive sites (e.g., Manaus). Species diversity is also high, often exceeding 225 species ha^−1^ [26], with an estimated mean above ground biomass of approximately 270 Mg ha^−1^ [27].

The first site, north of Manaus, the capital of Amazonas state, occupied an area of 5,042 km^2^ (2°33′11′′ S, 60°5′7′′ W, Figure 1) and included the Instituto Nacional de Pesquisas da Amazônia (INPA) and the Smithsonian Institution (SI) Biological Dynamics of Forest Fragments Project (BDFFP) research sites (established in 1979) [28], the Adolfo Ducke and Walter Egler forest reserves, and other state and federal environmental protection areas. Significant deforestation began in the area following construction in the early 1970s of the BR-174 highway connecting Manaus with Boa Vista in Roraima. Most deforestation activity occurred either side of the highway, with this fuelled primarily by agricultural expansion. Recognition of the opportunity to study the impacts of clearance and forest fragmentation on biodiversity as well as ecosystem services spurred the preservation of fragments of various dimensions (typically less than 2 km^2^) prior to felling of the surrounding tropical forest. Many of these fragments were, however, reconnected because of rapid regrowth of forests following abandonment of many clearances from the mid-1980s onwards. Up until the present, a substantial amount of research has been conducted, with these informing future management strategies across the Amazon [28].

The second site (3°10′5′′ S, 54°55′42′′ W) covered an area of 1,118 km^2^ and was located approximately 80 km to the south of Santarém, the second main city in the Pará state (Figure 1). This study area is within the Tapajós National Forest, between the Tapajós River and the BR-163 highway connecting Santarém to the state capital of Mato Grosso, Cuiabá. This conservation unit was created in 1974 and has been recognized as a model for sustainable forest management, including logging activities, at levels ranging from individuals to communities (e.g., [29], [30]). Compared to Manaus, forests are less dense with a mixed arrangement of broadleaves and palm trees such as Açaí (*Euterpe oleracea*) and Babaçu (*Orbignya phalerata*) being commonplace [31].

The third site (9°32′56′′ S and 62°6′27′′ W) occupied an area of 1,780 km^2^ and was located mostly within the Machadinho d’Oeste municipality, Rondônia state. This municipality originated from the former Machadinho settlement project, deployed by the Brazilian federal government (through the *Instituto Nacional de Colonização e Reforma Agrária*, INCRA) in 1982 as part of the POLONORDESTE Program [32]. As such, the majority of its inhabitants are dependent on agriculture for subsistence [33]. Open rain forest is the dominant vegetation type in the municipality [32]. According to data from the Brazilian Ministry of the Environment (MMA), this site includes several conservation units, mainly extractive reserves, which were established in and after 1995.

The area of the Manaus site was approximately three to four times the size of the other two study areas for several reasons. Deforestation in Manaus started in the early 1970s and land conversion from primary forest to agriculture/pasture was much more scattered. By comparison, deforestation patterns were more concentrated at Santarém (either side of and in proximity to a highway) and Machadinho d’Oeste (a planned settlement). A larger area was also selected to encompass older deforestation within the BDFFP study area and more recent clearing towards the city of Manaus, thereby allowing a wide range of LULCC processes to be captured.

At all three sites, much of the history of deforestation and land use has been captured by optical (primarily Landsat) sensors during the dry season, when cloud cover is minimal. In Manaus, a moderately strong dry season occurs between June and October [28], in Santarém between May and October [34], and in Machadinho d’Oeste between April and November [32]. Rainfall is generally higher at Manaus (between 1,900 and 2,500 mm annually) and lower at Santarém and Machadinho d’Oeste with an annual average of 2000 mm. The mean annual temperatures at the sites range from 25°C to 26°C [32], [34], [35]. The distribution of forests types is determined in part by the topography and geology. The topography is moderately flat (up to 160 m elevation) north of Manaus and is divided by a large number of waterlines. Soils are nutrient-poor (i.e., Oxisols), with these being typical of many across the Amazon basin [36]. The area south of Santarém is also moderately flat, ranging from approximately 50 to 240 m, and consists of nutrient poor soils (i.e., Oxisols and Ultisols) [34], [37]. The terrain is moderately undulated at Machadinho d’Oeste, ranging from approximately 90 to 370 m, and the dominant soil types are Alfisols, Oxisols, Ultisols, and Alluvial [32].

## Data

For Manaus, Landsat Multi-spectral Scanner (MSS), Thematic Mapper (TM) and Enhanced Thematic Mapper Plus (ETM+) data were acquired between 1973 and 2011 (path 231, row 62; Table 1). The Landsat MSS data acquired between 1973 and 1983 were only available as hard-copy prints [38]. The remaining Landsat TM and ETM+ scenes (1985 onwards) were provided in digital format from both INPE and the United States Geological Service (USGS).

**Table 1:** Landsat Multi-spectral Scanner (MSS), Thematic Mapper (TM) and Enhanced TM (ETM+) data available for the three Amazonian sites.

Manaus (Path 231, Row 62)		Santarém (Path 227, Row 62)		Machadinho d’Oeste (Path 231, Row 67)	
Date^1^	Sensor	Date^1^	Sensor	Date^1^	Sensor
19730707	MSS	19840824	TM	19840617	TM
19770731	MSS	19850726	TM	19860810	TM
19780822	MSS	19860729	TM	19870712	TM
19790703	MSS	19870716	TM	19890717	TM
19830709	MSS	19880803	TM	19900618	TM
19850604	TM	19890822	TM	19910925	TM
19880815	TM	19900809	TM	19940816	TM
19890802	TM	19910711	TM	19950803	TM
19910808	TM	19931020	TM	19960704	TM
19920607	TM	19951010	TM	19970723	TM
19941019	TM	19960825	TM	19980624	TM
19950920	TM	19970727	TM	19990729	TM
19960720	TM	19980815	TM	20010803	TM
19990713	TM	19990903	TM	20030724	TM
20010827	ETM+	20000905	TM	20050713	TM
20020830	ETM+	20010916	ETM+	20060716	TM
20030809	TM	20030829	TM	20070703	TM
20060716	TM	20050701	TM	20080806	TM
20070804	TM	20060805	TM	20090809	TM
20080806	TM	20070621	TM	20100625	TM
20090910	TM	20081130	TM	20110612	TM
20100727	TM	20090712	TM		
20110831	TM	20100629	TM		

1 Date format is yyyymmdd.

In the previous study of Prates-Clark et al., 2009 [38], scenes acquired between 1985 and 2003 were used to study forest regeneration in this area. In this study, the time-series was extended to 2011 using six additional annual scenes, which were relatively cloud free (<20%), or cloud was distributed in only certain areas of the image. In total, the time-series extended over an approximate 40-year period from 1973 (when the deforested area was minimal) to 2011. For Santarém, Prates-Clark et al., 2009 [38] again used Landsat TM and ETM+ imagery acquired between 1984 and 2003 (path 227, row 62), with most scenes unaffected by substantive cloud cover. The time-series was extended using Landsat TM data acquired for each year from 2005 to 2010. Coverage for 2011 was actively sought but no data with minimal cloud coverage were available. Whilst most changes at Santarém were associated with deforestation for agriculture and pasture, extensive wildfires damaged the surrounding forests in 1992 and 1997. The last fire episode was associated with the 1997–1998 El Niño Southern Oscillation (ENSO) event. The time-series classification for Machadinho d’Oeste had not been undertaken previously and so 21 new time-series digital images acquired between 1984 and 2011 were obtained from the USGS. Gaps in the time-series ranged from between one (70%) to approximately three years (5%). Cloud cover was minimal on all but two dates, although the vast majority of the deforested area was visible within these.

## Methods

### 4.1. Image pre-processing

Landsat images acquired by the USGS were orthorectified and calibrated to units of spectral radiance (W m^−2^ sr^−1^ µm^−1^) and then calibrated to top of atmosphere (TOA) reflectance using calibration factors and equations provided by Chander et al., 2009 [39]. Images acquired by INPE were processed to TOA reflectance and geometrically corrected using Environment for Visualizing Images (ENVI) software (Exelis Visual Information Solutions, Boulder, CO, USA), a third order polynomial and a nearest neighbor transformation [38]. Each Landsat image was then subsetted to encompass the main area of deforestation and the intersection of all images in the time series.

### 4.2. Image classification

As with Prates-Clark et al., 2009 [38], each digital image within the time-series for each site was classified into mature forest (MF), non-forest (NF), and secondary forest (SF), with the second category including crops (herbaceous and woody) and pastures. Areas of open water were also mapped and a common mask was applied to all dates in the time-series. Several classification approaches were utilized with the intention of providing the best classification of the desired categories. At Manaus, and for digital images acquired before 2006, supervised classifications based on the minimum distance or maximum likelihood algorithms were applied (e.g., [40]), with regions of interest representing the main cover types defined around a number of target areas described on the basis of field observations or through reference to very high resolution data. For the Landsat TM images acquired between 2006 and 2011, an object oriented classification (e.g., [41]) was followed and involved a decision-rule classification applied within the eCognition software (Trimble Geospatial Imaging, Munich, Germany). The rule base method used data from the available Landsat sensor bands as well as data layers derived from spectral indices, namely the Normalized Difference Vegetation Index (NDVI) and Normalized Difference Water Index (NDWI). The classification of MF was refined with a cloud-free mask of the MF area obtained through classification of the most-cloud-free and recent image in the time-series. Images acquired on the 31st August, 2011, 29th June, 2010 and 12th June 2011 were used to generate the MF mask, on the assumption that the land cover type had remained as MF for the whole of the time-series. If MF was covered by cloud in earlier images of the time-series, this was subsequently classified as MF. At Manaus, SF were older compared to the other sites and the structure of the upper canopy of the regenerating forests led to a spectral response in the near and shortwave infrared channels that was similar to MF; hence, reference to images earlier in the time-series was necessary [18], [22], [42]. At Santarém, the Landsat data acquired between 1984 and 2003 were classified using a fuzzy logic approach that was applied to the original Landsat bands as well as derived fractional images (shade/moisture, vegetation and soil). Each pixel of the Landsat data acquired between 2005 and 2010 was classified into one of the three main land cover classes (described above) using the random forests machine learning classification algorithm (e.g., [43]) and training data. This algorithm has a number of options (e.g., [43]) that were tested for each date to obtain the best possible classifier. As with Santarém, all Landsat TM dates for Machadinho d’Oeste were classified using the random forests algorithm. In all cases, the objective was to obtain the best classification of NF, MF and SF for each year of observation.

### 4.3. Post-classification

The comparison of the time-series of classified images (as MF, NF, or SF) identified several cases where some pixels classified as SF or NF in a given date were classified as MF in the following date. As this sequence is not plausible, in-house Interactive Data Language (IDL) code (Exelis Visual Information Solutions, Boulder, CO, USA) was written to identify these cases by comparing the classification over two consecutive dates. Where SF or NF areas were classified as such in one year and as MF in the next, the MF class was reallocated to SF. These areas were not reclassified as NF because it was assumed that spectral confusion between NF and MF was unlikely. This procedure was similar to that undertaken by Roberts et al., 2002 [44], who also considered “disallowed transitions” between cover types.

### 4.4. Deforestation and regrowth rates

Annual rates of change from MF (or SF) to NF (deforestation) and from NF to SF (regeneration) were calculated for consecutive dates in the time-series using equations (1) and (2) [45]

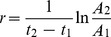
(1)

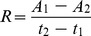
(2)where *r* and *R* are the annual rate of change expressed in percentage per year and hectares per year respectively; *A_1_* and *A_2_* are the MF (or SF, in the case of deforestation occurring in regeneration areas) or NF cover areas (hectares) at time *t_1_* and *t_2_* respectively (time period). These rates were used to characterize the temporal evolution of change across the three sites, to detect events (primarily clearance) and describe general trends.

### 4.5. Age of secondary forest, period of active land use, and frequency of clearance

Algorithms were written and implemented in IDL to compare the classifications of MF, NF, and SF between dates. Subsequently, datasets relating to the history and dynamics of land use, namely the age of secondary forest (ASF), the period of active land use (PALU) prior to abandonment, and the frequency of clearance (FC) were generated for each site and for each year in which an image had been acquired. The ASF was estimated by summing the time (in years) that each pixel was occupied by SF since the last clearance event. However, when SF occurs in the first date of the time series, and it is not subsequently cleared, the ASF can only be considered as a minimum age as the exact date of land abandonment is not known. The PALU was defined as the difference (in years) between the time of initial forest clearance and the onset of regeneration. However, where reclearance of regenerating forest had occurred, the PALU was calculated by summing the period since the last reclearance event until the forest cover had re-established. The FC was estimated by summing the frequency of transitions from MF (or SF) to NF. For both the Manaus and Santarém study areas, fire scars were evident within some clearances by the lower near and shortwave infrared reflectance compared to the original vegetated surface [46]. However, many burned areas acquired a vegetated cover quite rapidly [47], and so only a partial fire history could be retained.

To better spatially represent and discuss the main results, classes of ASF, PALU, and FC were generated and used to assess their temporal evolution. Each metric was classified into three classes as to identify a lower, middle and upper interval range. However, these intervals do have some degree of subjectivity. ASF classes were defined as initial (≤5 years), intermediate (6–15 years) and advanced (≥16 years), adapted from Lucas et al., 2000 [18]. PALU classes were defined as short (≤2 years), medium (3–4 years) and long (≥5 years), with these representing various different crop or pasture cycles. FC classes were defined as low (1 time), medium (2 times) and high (≥3 times), representing several land use patterns in terms of the frequency of the deforestation -> agriculture/pasture -> abandonment -> regrowth temporal sequence.

### 4.6. Accuracy assessment and area calibration of the time-series of classified images

The most recent very high resolution (VHR) imagery was used to carry out the accuracy assessment of the time-series classifications at each site. As VHR data were not available to perform the accuracy assessment for all the classified images in the time-series, the accuracy in the classification of the image that was closest in time was assumed to be similar for the remaining images. To quantify the accuracy of the 2010 classification for Santarém, reference was made to 5 m SPOT-5 panchromatic data from 2009 and 2011 whilst the 2007 and 2010 classifications for Manaus and Machadinho d’Oeste respectively were validated using GeoEye imagery available on Google Earth.

At each site, 200 random points located at the center of larger polygons (greater than 6 ha) were generated for each class. The points on the classification were compared with the VHR imagery from the same date. At Santarém, it was assumed that if the same land cover was present in both the 2011 and 2009 image, the land cover in 2010 would be the same. The accuracy assessment was reported as a standard error matrix, including the overall accuracy and omission and commission errors (e.g., [48]). From the same VHR imagery and past field studies of the study sites (e.g., [32], [38]), woody agriculture crops had established on some of the deforested areas. At Manaus, a large area of tree crops (TC, mainly oil palm) was progressively planted in the north west of the site from the 1983 to 1989, whilst at Machadinho d’Oeste, perennial crops (PC, mainly coffee plantations) were more common. To assess how well the areas of MF, NF and SF were discriminated using the classification, these areas were delineated manually from the VHR imagery and used in the generation of the error matrices.

The most straightforward way of estimating total areas for each class was to count the number of pixels in the MF, NF and SF classes with respect to the ground truth data. This is called “naïve estimation” in the remote sensing literature [49]. As a matter of fact, areal estimates obtained this way are biased (e.g., [50]). Some post-classification methods have been used to improve these estimates, namely the so-called calibration techniques that are divided into classical and inverse methods. The information contained in the error matrix was used to correct for misclassification bias [50]. Walsh and Burk, 1993 [50] carried out a simulation study and concluded that the inverse method consistently performed better than the classical method and lead to unbiased estimates for total areas. The idea behind this calibration technique was to use the misclassification probabilities among classes to revise the proportions given by pixel counting (*p_i_*) [51]. According to Tenenbein, 1972 [51] and Walsh and Burk, 1993 [50], the revised (calibrated) proportion of the total area occupied by class *i* (*π_i_*) can be estimated with equation (3)
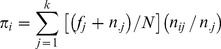
(3)where *i* represents the observed class, *j* the predicted class, *k* the number of classes, *n* the number of observations in the validation sample, *N* the combined number of observations in the validation and satellite datasets, *f_j_* the (*N*-*n*) in class *j*, *n_ij_* the number of observations in class *i* that were classified as *j*, and *n._j_* the sum of all training observations predicted as class *j*. This method was applied to calibrate the area estimates obtained from each date of classified images using the error matrices that were produced for the three sites.

## Results

### 5.1. Accuracy assessment of land cover classification

The error matrices resulting from the accuracy assessment over Manaus, Santarém, and Machadinho d’Oeste are presented in Table 2, 3 and 4 respectively. For all three sites, the overall accuracy was high (above 0.90), although some relatively high omission and commission errors were identified, especially in Santarém and Machadinho d’Oeste. For Manaus, the omission and commission errors were always below 10%. For Santarém, a higher omission error in the SF class was detected (17.0%), mainly due to misclassification as MF (11.5%), which reflects also in the high commission error in the MF class (10.5%). At Machadinho d’Oeste, a major source of error also comes from a high omission error in the SF class (28%), with this mainly being a consequence of misclassification as NF (18.5%) and to a lesser extent as MF (9.5%); a high commission error was also observed in the NF class (15.8%), with this being due to misclassification with SF.

**Table 2:** Error matrix obtained from the accuracy assessment of the 2007 land cover map of the Manaus site (MF – mature forest, NF – non-forest, SF – secondary forest).

	Classified				
Reference	MF	NF	SF	Total	Omission error
**MF**	186	4	10	200	0.070
**NF**	8	185	7	200	0.075
**SF**	11	2	187	200	0.065
**Total**	205	191	204	600	
**Commission error**	0.093	0.031	0.083		

The overall accuracy was 0.930.

**Table 3:** Error matrix obtained from the accuracy assessment of the 2010 land cover map of the Santarém site (MF – mature forest, NF – non-forest, SF – secondary forest).

	Classified				
Reference	MF	NF	SF	Total	Omission error
**MF**	196	0	4	200	0.020
**NF**	0	198	2	200	0.010
**SF**	23	11	166	200	0.170
**Total**	219	209	172	600	
**Commission error**	0.105	0.053	0.035		

The overall accuracy was 0.93.

**Table 4:** Error matrix obtained from the accuracy assessment of the 2010 land cover map of the Machadinho d’Oeste site (MF – mature forest, NF – non-forest, SF – secondary forest).

	Classified				
Reference	MF	NF	SF	Total	Omission error
**MF**	200	0	0	200	0.000
**NF**	0	197	3	200	0.015
**SF**	19	37	144	200	0.280
**Total**	219	234	147	600	
**Commission error**	0.087	0.158	0.020		

The overall accuracy was 0.90.

In Manaus, TC (4,774 ha) was mostly incorrectly classified as SF (89%), illustrating the similarity in their spectral signatures, but accounted only for 4.4% of the SF area in 2007 (Table 5). However, most of the TC classified as SF in Manaus was associated with the oil palm plantation situated in the north west of the study site. The majority of TC in Machadinho d’Oeste was correctly classified as NF, although the overall area was very small (17 ha). Conversely, 70% of the PC (mainly coffee plantations) at Machadinho d’Oeste was misclassified as SF, although this only accounted for 2.1% the entire SF area in 2010.

**Table 5:** Area (ha) and relative incidence (RI, %) of perennial (PC) and tree (TC) crops with the mapped area of mature forest (MF), non-forest (NF) and secondary forest (SF).

	Manaus (2007)		Machadinho d’Oeste (2010)			
	TC		PC		TC	
Classified	Area (ha)	RI (%)	Area (ha)	RI (%)	Area (ha)	RI (%)
MF	139	0.0	31	0.1	0	0.0
NF	398	1.0	540	0.9	14	0.0
SF	4,237	4.4	1,323	2.1	3	0.0
Total	4,774		1,894		17	

Estimates were based on the land cover map closest to the date that was used to delineate crop types using very high resolution (VHR) imagery.

### 5.2. Land cover change

**Figure 2:**
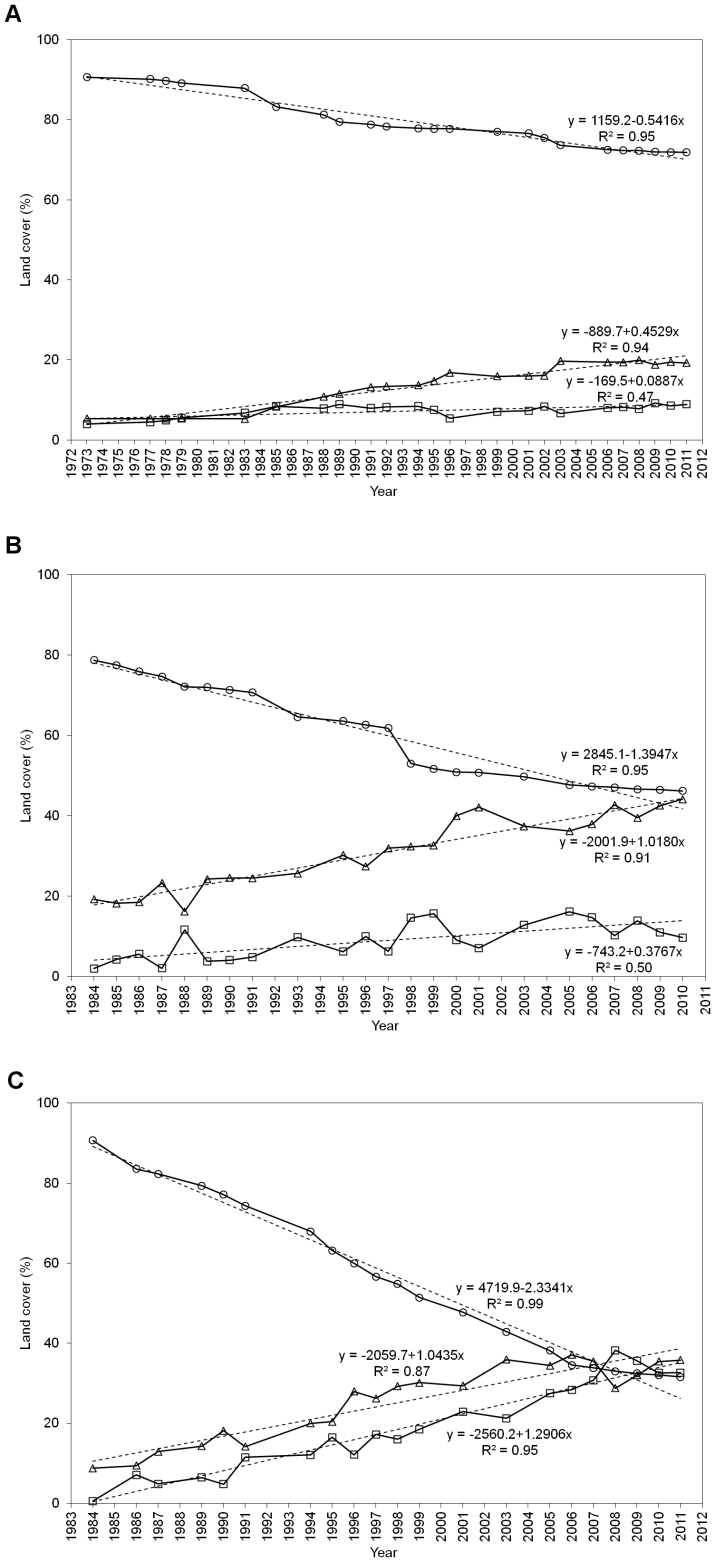
Land cover change (%) across the three selected sites. Land cover change (%) across the three selected sites; A) Manaus (1973–2011), B) Santarém (1984–2010), and C) Machadinho d’Oeste (1984–2011). Each dashed line represent the linear fit of the proportion of each land cover class as a function of the year; also showing are the corresponding equations and coefficient of determination (R^2^).(○ - mature forest, □ - non-forest, Δ- secondary forest).

The evolution of land cover change in Manaus, Santarém and Machadinho d’Oeste is depicted in Figure 2A, 2B and 2C respectively and in Table 6, which shows that the most rapid change occurred in Machadinho d’Oeste, followed by Santarém and Manaus. For all sites, there was a strong correspondence between the land cover percentage and time (year) for the MF class, with the rate decreasing by an average of 0.54% in Manaus to 1.39% in Santarém and 2.33% in Machadinho d’Oeste. For all sites, a corresponding increase in the area deforested occurred, with the land cover alternating between NF and SF in all cases. The change rate in the NF class increased from 0.09% (Manaus) and 0.38% (Santarém) to 1.29% (Machadinho d’Oeste) whilst the SF change rate progressively increased from Manaus (0.45%) to Santarém (1.02%) and Machadinho d’Oeste (1.04%). The relationships between the proportion (%) of MF, NF and SF and time (year) were significant at all sites (P<0.001).

**Table 6:** Land cover proportion (%) of mature forest (MF), non-forest (NF) and secondary forest (SF) in each year of the time-series at Manaus, Santarém and Machadinho d’Oeste.

Manaus				Santarém				Machadinho d’Oeste			
Year	MF	NF	SF	Year	MF	NF	SF	Year	MF	NF	SF
1973	91	4	5	1984	79	2	19	1984	91	1	9
1977	90	4	5	1985	78	4	18	1986	84	7	9
1978	90	5	5	1986	76	6	19	1987	82	5	13
1979	89	6	5	1987	75	2	23	1989	79	6	14
1983	88	7	5	1988	72	12	16	1990	77	5	18
1985	83	8	8	1989	72	4	24	1991	74	11	14
1988	81	8	11	1990	71	4	25	1994	68	12	20
1989	79	9	12	1991	71	5	24	1995	63	16	20
1991	79	8	13	1993	65	10	26	1996	60	12	28
1992	78	8	13	1995	64	6	30	1997	57	17	26
1994	78	8	14	1996	63	10	27	1998	55	16	29
1995	78	7	15	1997	62	6	32	1999	51	18	30
1996	78	5	17	1998	53	15	32	2001	48	23	29
1999	77	7	16	1999	52	16	33	2003	43	21	36
2001	77	7	16	2000	51	9	40	2005	38	27	34
2002	76	8	16	2001	51	7	42	2006	35	28	37
2003	74	7	20	2003	50	13	37	2007	34	31	35
2006	73	8	19	2005	48	16	36	2008	33	38	29
2007	72	8	19	2006	47	15	38	2009	32	36	32
2008	72	8	20	2007	47	10	43	2010	32	33	35
2009	72	9	19	2008	47	14	40	2011	32	33	36
2010	72	9	19	2009	46	11	43				
2011	72	9	19	2010	46	10	44				

### 5.3. Deforestation and regrowth rates

Tables depicting relative and absolute deforestation and regeneration rates between consecutive dates for Manaus, Santarém and Machadinho d’Oeste are presented as Supporting Information (Table S1, S2 and S3 respectively in File S1) and a summary is given in Tables 7, 8 and 9 respectively. The maximum relative annual rate of deforestation over MF was observed at Santarém, with a 15.6% yr^−1^ loss between 1997 and 1998, with this associated with the extensive wildfires in 1997, and the highest average annual rate of deforestation was in Machadinho d’Oeste (3.9% yr^−1^). The maximum relative rates of SF clearance were in Machadinho d’Oeste (88.1% yr^−1^, 1990–1991) and Santarém (82.3% yr^−1^, 1987–1988), with average rates being 19.5% and 16.7% for these sites respectively. The maximum and the highest average relative rates of regeneration were observed at Santarém, with 261.5% yr^−1^ (1997–1998) and 78.5% yr^−1^ respectively. The lowest relative rates of regrowth were at Machadinho d’Oeste, with these averaging 25.8% yr^−1^. The average overall relative deforestation rates (combined MF and SF) were greatest for Machadinho d’Oeste (7.2% yr^−1^), followed by Santarém (4.9% yr^−1^) and Manaus (1.4% yr^−1^). On average, deforestation rates over SF were higher than those over MF, indicating reclearance at all sites and particularly Machadinho d’Oeste (averaging 19.5% yr^−1^) and Santarém (averaging 16.7% yr^−1^).

**Table 7:** Summary of deforestation and regrowth rates between consecutive dates in the Manaus time-series (MF – mature forest; SF – secondary forest).

	MF annual rate of deforestation		SF annual rate of deforestation		Annual rate of deforestation		Annual rate of regrowth	
	(% yr^−1^)	(ha yr^−1^)	(% yr^−1^)	(ha yr^−1^)	(% yr^−1^)	(ha yr^−1^)	(% yr^−1)^	(ha yr^−1^)
Minimum	0.1	277	0.0	0	0.1	691	0.0	0
Maximum	2.9	13,451	27.7	8,453	3.8	17,936	133.9	24,738
Mean	0.7	3,123	6.4	3,231	1.3	6,355	34.1	6,017
Median	0.4	1,591	5.0	2,725	0.9	4,513	24.8	4,793
Inter-quartile range	0.4	2,172	5.1	3,006	1.3	5,881	31.9	6,156
Standard deviation	0.8	3,726	6.5	2,699	1.1	4,905	33.7	5,756

**Table 8:** Summary of deforestation and regrowth rates between consecutive dates in the Santarém time-series (MF – mature forest; SF – secondary forest).

	MF annual rate of deforestation		SF annual rate of deforestation		Annual rate of deforestation		Annual rate of regrowth	
	(% yr^−1^)	(ha yr^−1^)	(% yr^−1^)	(ha yr^−1^)	(% yr^−1^)	(ha yr^−1^)	(% yr^−1^)	(ha yr^−1^)
Minimum	0.4	235	0.5	194	0.5	448	10.5	832
Maximum	15.6	11,046	82.3	11,352	17.7	16,952	261.5	12,257
Mean	2.1	1,598	16.7	3,189	4.9	4,787	78.5	4,675
Median	1.2	963	10.7	2,769	4.0	4,236	71.3	3,361
Inter-quartile range	1.3	1,085	16.6	2,350	3.0	2,831	79.6	4,031
Standard deviation	3.2	2,292	18.7	2,849	4.4	4,188	61.5	3,332

**Table 9:** Summary of deforestation and regrowth rates between consecutive dates in the Machadinho d’Oeste time-series (MF – mature forest; SF – secondary forest).

	MF annual rate of deforestation		SF annual rate of deforestation		Annual rate of deforestation		Annual rate of regrowth	
	(% yr^−1^)	(ha yr^−1^)	(% yr^−−1^)	(ha yr^−1^)	(% yr^−1^)	(ha yr^−1^)	(% yr^−1^)	(ha yr^−1^)
Minimum	1.2	748	0.0	17	1.5	2,386	3.7	103
Maximum	9.9	9,223	88.1	16,711	17.7	18,202	84.2	17,389
Mean	3.9	4,142	19.5	5,289	7.2	9,286	25.8	6,804
Median	3.5	4,171	14.3	4,273	6.0	7,436	19.1	6,172
Inter-quartile range	3.4	4,057	13.5	7,114	6.7	9,571	18.0	4,609
Standard deviation	2.3	2,394	19.5	4,467	4.4	5,248	21.5	4,141

Absolute areas (annual or based on periods of observation) are useful when comparing deforestation and regeneration rates between sites and the processes of change. Several key indicators are then listed in Table 10 together with the dates for each site to which these apply. For all sites, a trend from indicators a) to f) was observed with the majority being cleared initially, regenerating forests progressively establishing with these then recleared to varying degrees, and, of these, several are then abandoned to regrowth again.

**Table 10:** An assessment of the dynamics of land use at Manaus, Santarém and Machadinho d’Oeste (figures in brackets represent the number of years).

		Sites		
Indicators	Impact	Manaus	Santarém	Machadinho d’Oeste
a) Clearance of MF only with little or nodeforestation over SF	High pressure for new land	1973–1985 (12)	Pre-1984	1984–1987 (3)
b) Area of MF cleared>SF cleared.	Pressure for new land withsome containedre-use of the existing deforestedarea (through reclearance)	1985–1989 (4)	1986–1987 (1)	1987–1990 (3)
		2002–2003 (1)	1991–1993 (2)	1991–1996 (5)
			1997–1998 (1)	1997–1998 (1)
			2006–2007 (1)	2001–2003 (2)
			2008–2009 (1)	
c) Area of MF cleared<SF cleared	Greater re-use of existing deforested landbut still requirement for more land	1989–2002 (13)	1984–1986 (2)	1990–1991 (1)
		2003–2011 (11)	1987–1991 (4)	1996–1997 (1)
			1993–1997 (4)	1998–2001 (3)
			1998–2006 (8)	2003–2011 (8)
			2007–2008 (1)	
			2009–2010 (1)	
d) Area of MF and SF cleared>area ofSF regenerating		1973–1985 (12)	1984–1986 (2)	1984–1986 (2)
		1988–1989 (1)	1987–1988 (1)	1987–1989 (2)
		1991–1994 (3)	1989–1993 (4)	1990–1995 (5)
		1996–2002 (6)	1995–1996 (1)	1996–1997 (1)
		2003–2007 (4)	1997–1999 (2)	1998–2001 (3)
		2008–2009 (1)	2001–2005 (4)	2003–2008 (5)
		2010–2011 (1)	2007–2008 (1)	2010–2011 (1)
e) Reclearance of SF but no clearance of MF	Contained re-use of area already deforested	No years	No years	No years
f) Area of SF regeneration>MF andSF deforestation	Net abandonment of land to regenerating forests	1985–1988 (3)	1986–1987 (1)	1986–1987 (1)
		1989–1991 (2)	1988–1989 (1)	1989–1990 (1)
		1994–1996 (2)	1993–1995 (2)	1995–1996 (1)
		2002–2003 (1)	1996–1997 (1)	1997–1998 (1)
		2007–2008 (1)	1999–2001 (2)	2001–2003 (2)
		2009–2010 (1)	2005–2007 (2)	2008–2010 (2)
			2008–2010 (2)	

### 5.4. Age of secondary forest (ASF), period of active land use (PALU), and frequency of clearance (FC)

**Figure 3:**
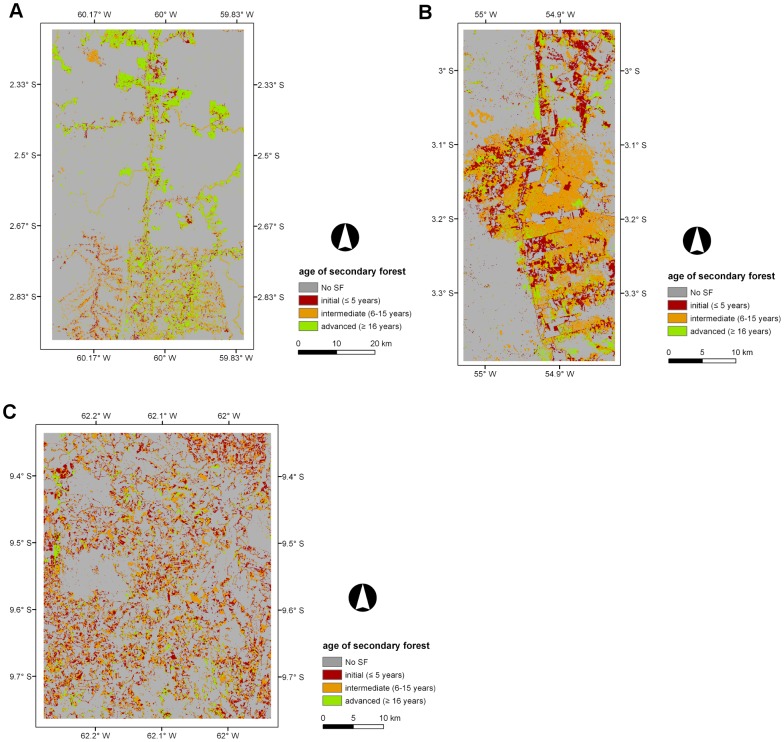
Maps displaying the age of secondary forest (ASF, years) across the three selected sites. Maps displaying the age of secondary forest (ASF) across the three selected sites for areas undergoing secondary forest (SF) in the last year of the corresponding time-series; A) Manaus (2011), B) Santarém (2010), and C) Machadinho d’Oeste (2011).

**Figure 4:**
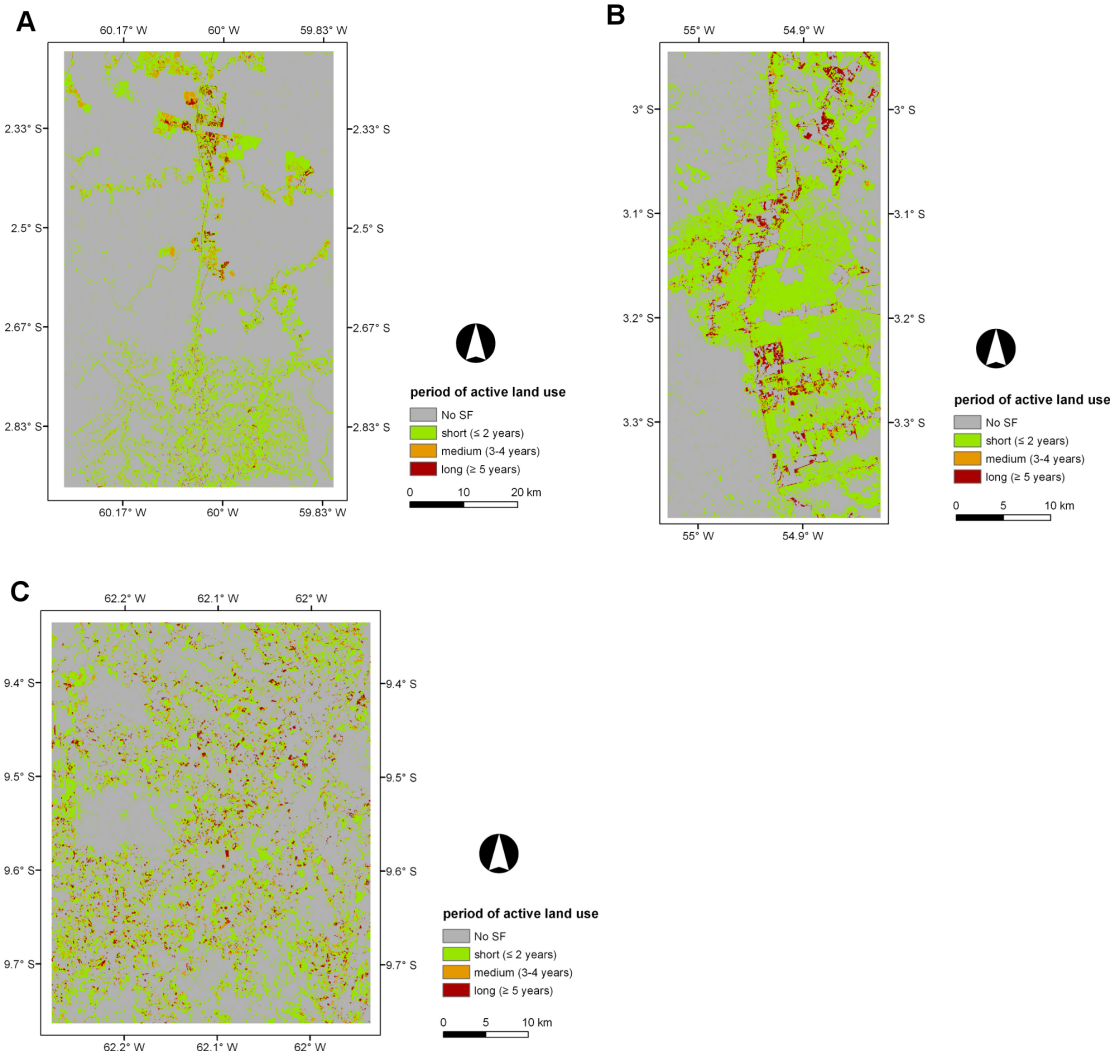
Maps displaying the period of active land use (PALU, years) across the three selected sites. Maps displaying the period of active land use (PALU, years) across the three selected sites for areas undergoing secondary forest (SF) in the last year of the corresponding time-series; A) Manaus (2011), B) Santarém (2010), and C) Machadinho d’Oeste (2011). An area of 667 ha (1.5%) with a PALU of zero in the Santarém site was included in the short PALU class (≤2 years) and corresponds to areas that were SF from 1984 to 2010.

**Figure 5:**
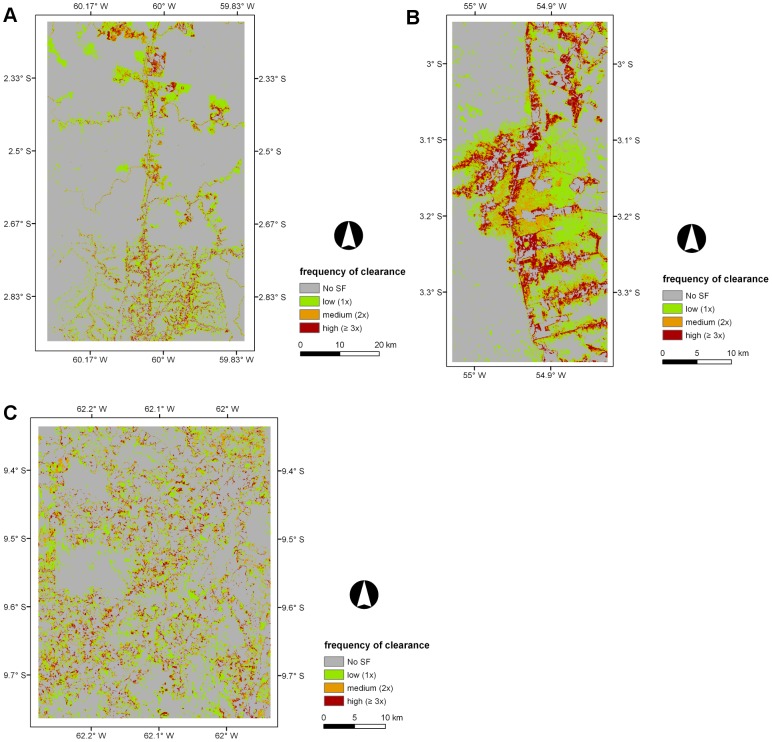
Maps displaying the frequency of clearance (FC) across the three selected sites. Maps displaying the frequency of clearance (FC) across the three selected sites for areas undergoing secondary forest (SF) in the last year of the corresponding time-series; A) Manaus (2011), B) Santarém (2010), and C) Machadinho d’Oeste (2011). In Manaus, Santarém and Machadinho d’Oeste an area of 103 ha (0.1%), 726 ha (1.6%), and 55 ha (0.1%), respectively, with a PALU of zero was included in the low FC class (1 time) and corresponded to areas that were non-forest (NF) in the first date of the time-series and no clearance has occurred in the secondary forest (SF) that persisted until the end of the time-series. In Santarém, the 726 ha also included areas that were already SF in 1984 that persisted until 2010.

**Figure 6:**
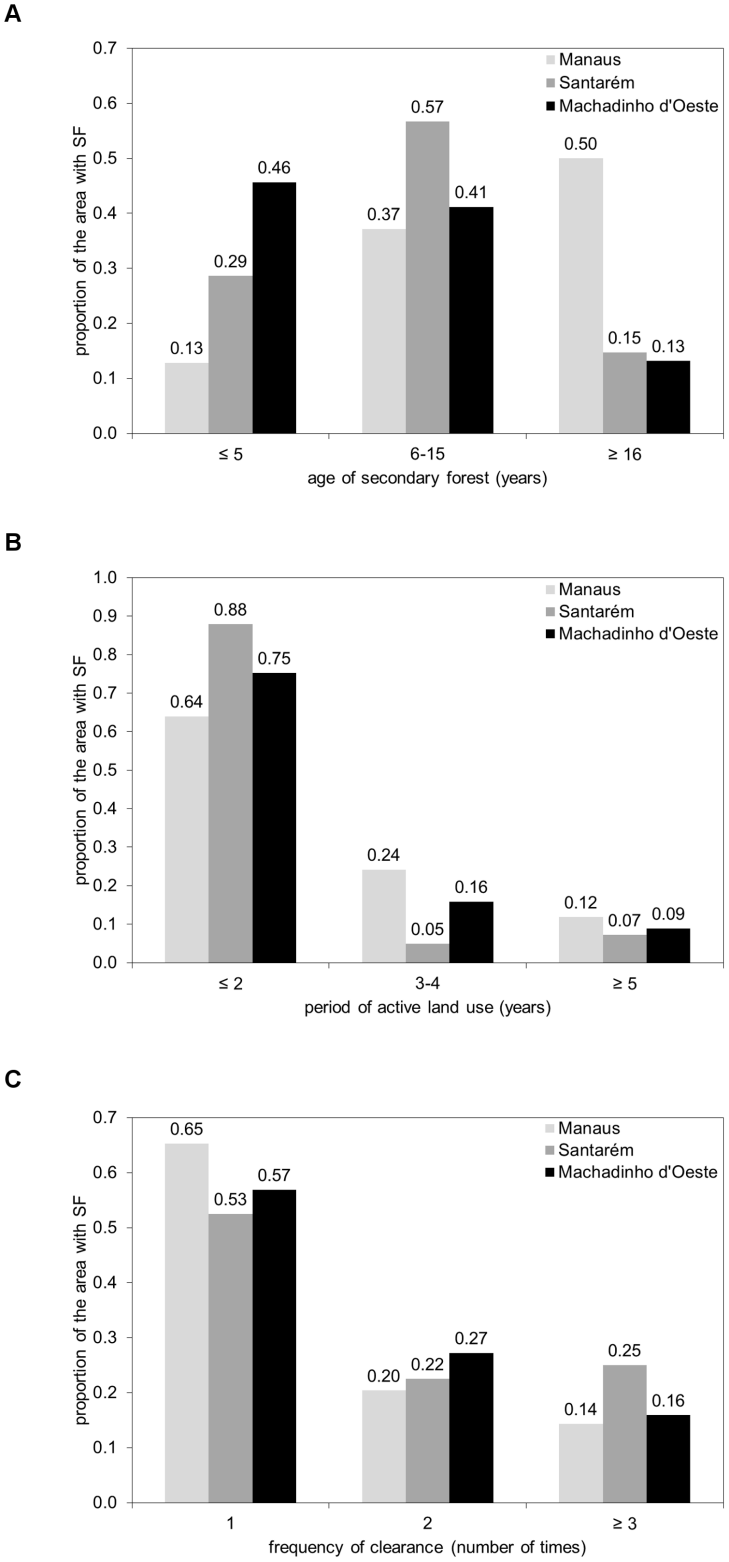
Classes of age of secondary forest, period of active land use, and frequency of clearance for the three sites. Classes of A) age of secondary forest (ASF), B) period of active land use (PALU), and C) frequency of clearance (FC) for the three sites, as a proportion of the area with secondary forest (SF) in the last date of the time-series. The proportion of the area with SF in the first class of PALU (≤2 years) in the Santarém site includes a small proportion (1.5%) corresponding to areas of SF persisting in all dates of the time-series. The proportion of the area with SF in the first class of FC (1x) in Manaus, Santarém and Machadinho d’Oeste includes a small percentage (0.1%, 1.6%, 0.1%, respectively) corresponding to those cases where non-forest (NF) was observed in the first date of the time-series and no clearance has occurred (i.e., the only transition was from NF to SF).

Maps of the ASF, PALU, and FC for the last date of the time-series are presented in Figures 3, 4 and 5 for Manaus (2011), Santarém (2010) and Machadinho d’Oeste (2011) respectively and the proportion of areas with respect to these classes is indicated in Figure 6. At Manaus, regrowth forests were comparatively older, with 50% occupied by forests ≥16 years (Figure 6A). At Santarém, 57% of forests were aged 6–15 years, and at Machadinho d’Oeste, the majority of the area occupied by SF aged ≤5 years (46%). Hence, the three sites contain different distributions of age classes, with this attributable to differences in the time since deforestation but also the PALU and the frequency of clearance events. At all sites, the majority of land has been used actively for ≤2 years (Figure 6B), either following the initial clearance of the primary forest or subsequent reclearance events, but the deforestation had commenced at different times. At Manaus, the PALU was also ≤2 years for over 64% of the area and many of the forests had been able to regenerate undisturbed, with some approaching 40 years in 2011, and had been cleared mainly on one (65%) occasion (Figure 6C). At Santarém, 88% of the area under regrowth had been used actively for ≤2 years prior to the last abandonment and the majority (53%) cleared only once. At Machadinho d’Oeste, the PALU was typically ≤2 years (75%), and again, forests were relatively young because most of the clearance occurred only on one (57%) occasion. Hence, different typologies of SF were observed at each site.

As a result of combining the 3-class PALU and FC maps for the last date of the time-series, a proxy for land use intensity in each site was generated (Table 11). At Manaus, of the 65% of land occupied by SF in 2011 that was subjected to only one clearing event, 41% had a short PALU but a significant proportion (16% and 9%) had a medium and long PALU respectively. Santarém is paradigmatic, as almost all land with SF in 2010 that was cleared only once was subjected to a short PALU. Assuming that a high FC and short and medium PALU will represent a higher land use intensity, then Manaus had 13% of the area undergoing SF in 2011 in these conditions, while Santarém and Machadinho d’Oeste had 20% and 15% in 2010 and 2011 respectively.

**Table 11:** Percentage of area with SF in the last date of the time-series, per combination of PALU and FC classes.

PALU (classes)	FC (classes)			Total
**Manaus**	**Low (1x)**	**Medium (2x)**	**High (≥3x)**	
Short (≤2 years)	41	13	10	64
Medium (3–4 years)	16	5	3	24
Long (≥5 years)	9	2	1	12
**Total**	66	20	14	
**Santarém**	**Low (1x)**	**Medium (2x)**	**High (≥3x)**	
Short (≤2 years)	51	20	17	88
Medium (3–4 years)	1	1	3	5
Long (≥5 years)	0	2	5	7
**Total**	52	23	25	
**Machadinho d’Oeste**	**Low (1x)**	**Medium (2x)**	**High (≥3x)**	
Short (≤2 years)	48	17	11	75
Medium (3–4 years)	5	7	4	16
Long (≥5 years)	4	4	1	9
**Total**	57	28	16	

**Figure 7:**
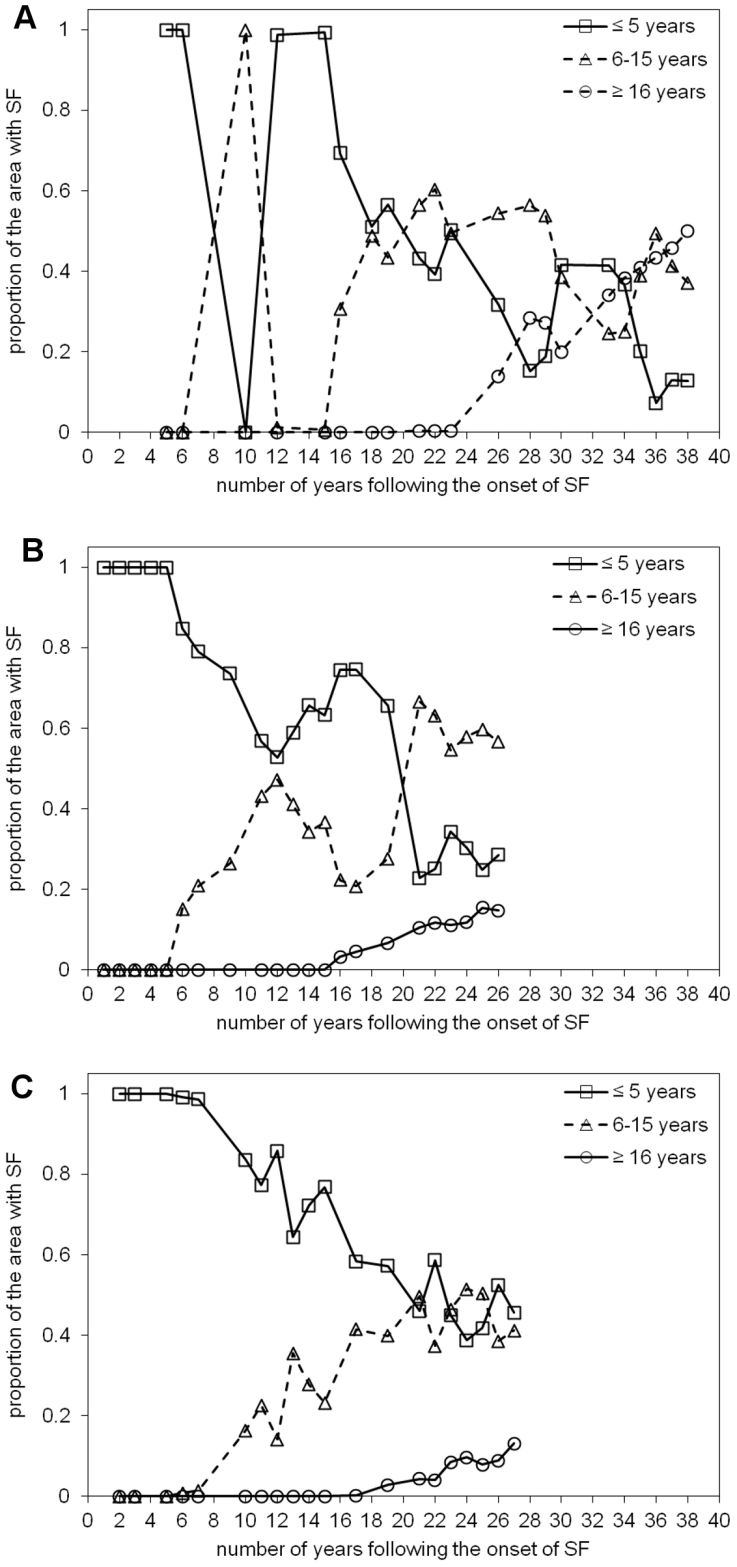
Proportion of the area with secondary forest (SF) as a function of the number of years following the onset of SF for the three classes of age of secondary forest. Proportion of the area with secondary forest (SF) as a function of the number of years following the onset of SF for the three classes of age of secondary forest (ASF) in A) Manaus, B) Santarém, and C) Machadinho d’Oeste: initial, ≤5 years; intermediate, 6–15 years; advanced, ≥16 years.

To investigate whether these typologies were dependent on the last date of the time-series, the same analysis was performed for all the dates in the time-series. At Manaus, the proportion of the area with SF in the ≤5 year ASF class decreased irregularly with the onset of regeneration up to approximately 15% in the last date of the time-series (Figure 7A), with this pattern being mirrored for the 6–15 year ASF class, and the ≥16 year ASF class steadily increasing up to around 50% in 2011. At Santarém (Figure 7B), the proportion of the area with SF in the initial ASF class decreased to around 30% in 2010, again mirrored by an overall increase to about 60% in the 6–15 year ASF class. As with Manaus, the proportion of the area with SF in the advanced ASF class increased from 15 years following the onset of regeneration to cover approximately 15% in 2010. In Machadinho d’Oeste (Figure 7C), the proportion of SF in the initial ASF class systematically decreased from the onset of regeneration leveling at around 45% in 2011, and an opposite pattern was observed in the intermediate ASF class, with a systematic increase since the onset of regeneration up to approximately 40% in 2011. As in Manaus and Santarém, a steady increase in the proportion of area with regeneration in the advanced ASF class was observed, from 17 years after the onset of regeneration up to around 15% in 2011. By combining the initial and intermediate ASF classes, a more consistent but asymptotic increase in the area of SF was observed, with this compensating for the interplay between these ASF classes, which occurs because of reclearance. The general trend across all sites is a decrease in the proportion of the area with SF in the initial ASF class up to the present, with a simultaneous increase in the proportion of the intermediate and advanced ASF classes. No asymptotic pattern is visible in the relationship between the proportion of the area with SF and the number of years following the onset of regeneration for this variable in the three selected sites.

**Figure 8:**
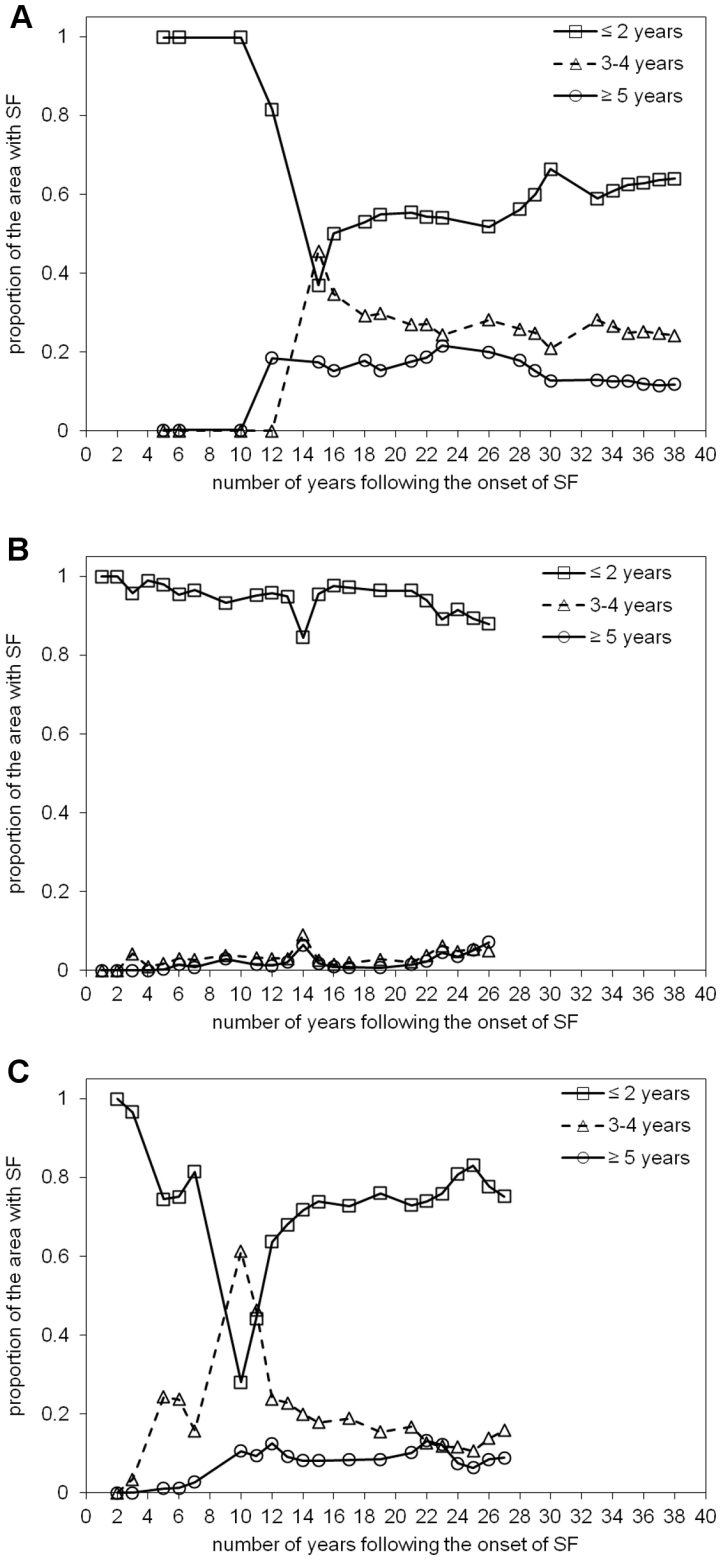
Proportion of the area with secondary forest (SF) as a function of the number of years following the onset of SF for the three classes of period of active land use. Proportion of the area with secondary forest (SF) as a function of the number of years following the onset of SF for the three classes of period of active land use (PALU) in A) Manaus, B) Santarém, and C) Machadinho d’Oeste: short, ≤2 years; medium, 3–4 years; long, ≥5 years.

At Manaus, the proportion of areas with a short PALU was initially high (100%) but, over time, this area decreased to about 40% but then increased subsequently to 60%, where it remained constant 32 years from the onset of regeneration (Figure 8A). This decrease was associated with an increase in the area with a medium and long PALU (14–16 years after the onset of SF regeneration). At Santarém (Figure 8B), the proportion with short PALU remained high, with this suggesting clearance of SF was common practice. The areas with medium and long PALU remained relatively low (<5–10%) but increased slightly towards the end of the time-series. At Machadinho d’Oeste, the temporal pattern was similar to that observed at Manaus; after 12–14 years, the proportion of short, medium and long PALU stabilized around 75%, 15% and 10% respectively.

**Figure 9:**
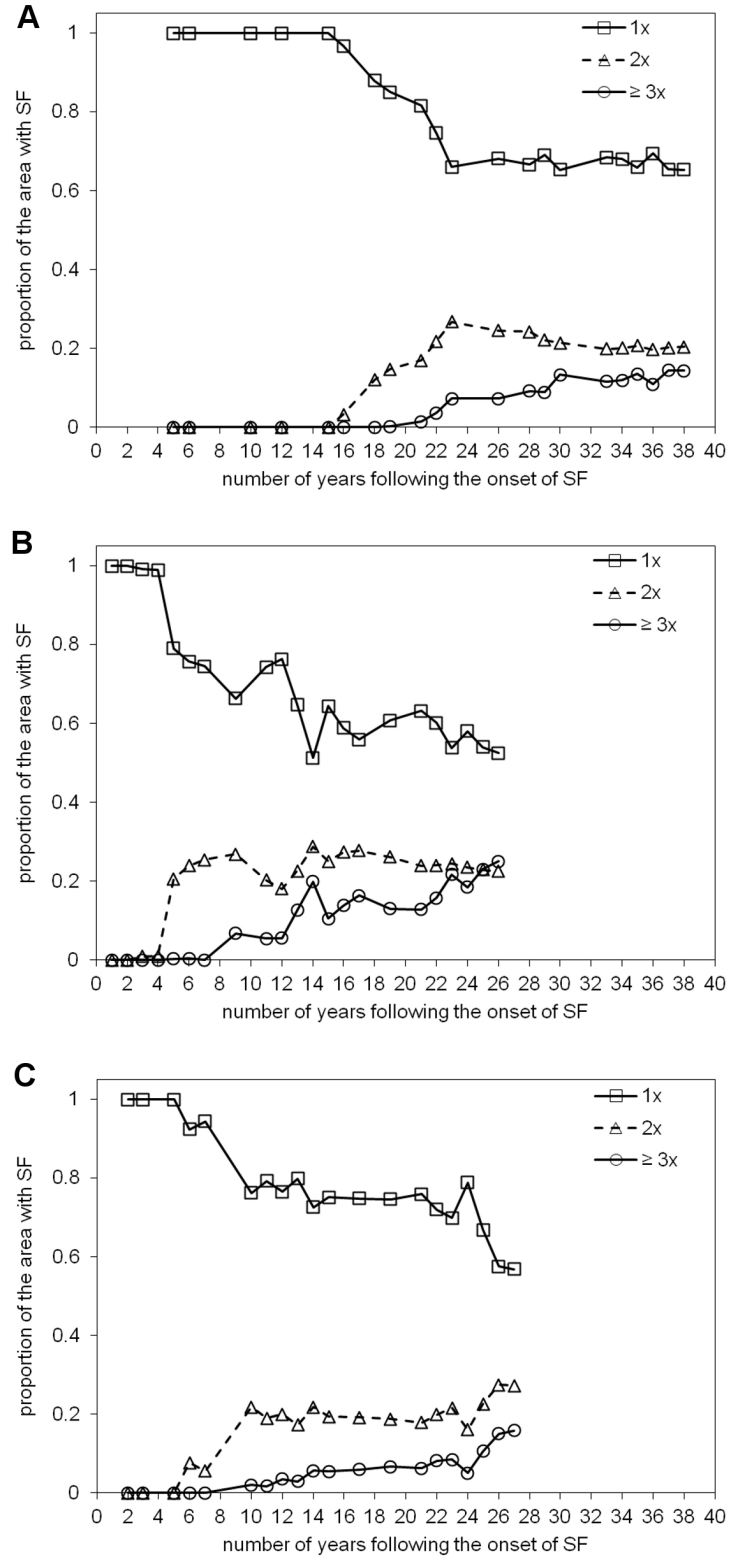
Proportion of the area with secondary forest (SF) as a function of the number of years following the onset of SF for the three classes of frequency of clearance. Proportion of the area with secondary forest (SF) as a function of the number of years following the onset of SF for the three classes of frequency of clearance (FC) in A) Manaus, B) Santarém, and C) Machadinho d’Oeste: low, 1 time; medium, 2 times; high, ≥3 times.

The FC over areas undergoing SF also varied between sites (Figure 9). At Manaus, the SF had been cleared only once until about 14 years after the onset of SF, but this proportion decreased, stabilizing at approximately 65% in 2011. This occurred because SF began to be cleared again 15 years after the onset of regeneration and again after 19 years (i.e., 3 FC events), with these areas stabilizing at approximately 20% and 15% respectively. At Santarém, SF was cleared within 2–4 years from the onset of regeneration and continually thereafter. This was reflected in the increase in the proportion of SF areas cleared twice (after 4 years) and three times (after 7 years) with these both stabilizing at approximately 20%. At Machadinho d’Oeste, the proportion of areas with only a clearing event decreased to 55% 28 years after the onset of regeneration. The medium and high FC classes commenced after 4 years and 7 years respectively with the proportions of these remaining relatively constant (25% and 15%).

## Discussion

### 6.1. Accuracy assessment of the land cover time-series

Several studies carried out in the Amazon have acknowledged some issues in discriminating SF from MF and, sometimes, NF. Lucas et al., 2000 [18] reported omission and commission errors ranging from 55–75% and 51–76%, respectively, associated with a greater confusion between NF and SF rather than between MF and SF. Carreiras et al., 2006 [20] reported an omission and commission error in the SF class of 66% and 43%, respectively, due to confusion between the SF, MF and NF classes. These authors concluded that misclassification among SF, MF, and NF was understandable, since from a spectral standpoint, SF is a transitional class between NF (i.e., agriculture/pasture) and MF. The initial stages of regeneration are spectrally similar to agriculture/pasture. Conversely, final stages of regrowth are more related to MF. The values reported in the two previous studies are much higher than those from this study, and that could be associated with the spatial resolution of the datasets that were used: ∼1-km SPOT 4 VEGETATION and NOAA AVHRR vs. the 30-m spatial resolution Landsat TM and ETM+ data. The spatial arrangement of SF in the BLA, dominated by small previously abandoned areas, especially in Rondônia (e.g., [52]), creates an additional difficulty when trying to discriminate these from the surrounding land cover classes with coarse spatial resolution data. Kimes et al., 1999 [53] used 20-m spatial resolution SPOT High Resolution Visible (HRV) data between 1986 and 1994 to discriminate MF, NF and SF in Rondônia and reported an accuracy of 95% in the SF class with misclassification happening mostly with the MF class. Metzger, 2002 [54] used Landsat TM data over the Bragantina region (Pará state) in 1996 to map various land cover classes and reported omission and commission errors in SF age classes ranging from 8–11% and 7–10%, respectively, with misclassification occurring mainly from confusion with MF. Vieira et al., 2003 [55] used Landsat 7 ETM+ data to map different stages of SF in São Francisco do Pará (northeastern Pará state) and reported omission and commission errors between 17–25% and 22–40% respectively, due mainly to misclassification among SF stages. Kuplich, 2006 [56] used Landsat TM and Synthetic Aperture Radar (SAR) data to discriminate various land cover classes in Manaus (included the BDFFP) and reported omission and commission errors in the SF age classes of 10–99% and 32–80% respectively, but those errors decreased when the SF age classes were aggregated into a unique class (78% and 25%, respectively). Although errors are still high in some studies (e.g., [18], [20], [56]), others have been able to discriminate SF from MF and NF with a high degree of accuracy (e.g., [53]–[55]).

Prates-Clark et al., 2009 [38] validated the 1995 and the 2001 land cover maps over Manaus and Santarém respectively using field-based reference data. For the validation of the 1995 land cover map over Manaus, field data collected in 1995 were used [22], [42] and the authors reported an overall accuracy of ∼99%, with omission errors in the MF, NF and SF classes of 0.3%, 3.5% and 1.9%, respectively, and commission errors of 0.5%, 7.0% and 9.5%, respectively. The major source of error in the SF class (higher commission error) was misclassification as MF class. At Santarém, field data collected in 2002 [57] were used to validate the 2001 land cover map. The overall accuracy was 88%, the omission errors were 2.2%, 1.1% and 4.2% for the MF, NF and SF classes respectively, and the commission errors were 0.6%, 28.8% and 71.9% respectively. The particularly high commission error in the SF class was essentially the consequence of misclassification as MF. The NF class displayed a high commission error that was related to misclassification as both MF and SF. Higher errors indicated in Prates-Clark et al., 2009 [38] for the Santarém site were also depicted in our accuracy assessment of the 2010 land cover map, although of much lower magnitude.

A major limitation of the accuracy assessment carried out in this study it that only one date (classification) of each time-series per study area was validated with very high spatial resolution data. Nevertheless, a 10-fold cross-validation procedure was implemented for those dates classified with the random forests algorithm, i.e., the 21 Landsat TM images of the Machadinho d’Oeste time-series (1984–2011) and the post 2003 Santarém Landsat TM images (2005–2010). In Machadinho d’Oeste, the average overall accuracy was ∼98%, the omission errors of the MF, NF and SF classes ranged from 0.0–4.4%, 0.0–2.1% and 0.7–7.6% respectively, and the commission errors were between 0.0–4.2%, 0.0–0.3% and 1.7–7.1% respectively. At Santarém, the 2005–2010 time-series of classified Landsat TM data resulted in an average overall accuracy of 95%, the omission errors of the MF, NF and SF classes ranged between 1.8–6.7%, 0.0–0.9% and 8.1–16.7%, respectively, and the commission errors were between 3.7–7.5%, 0.0–0.6% and 4.1–13.2% respectively. Both the omission and commission errors of the NF class were very low, thus indicating that the major source of error of the SF class was misclassification as MF.

### 6.2. Differences of land cover change patterns among sites

**Figure 10:**
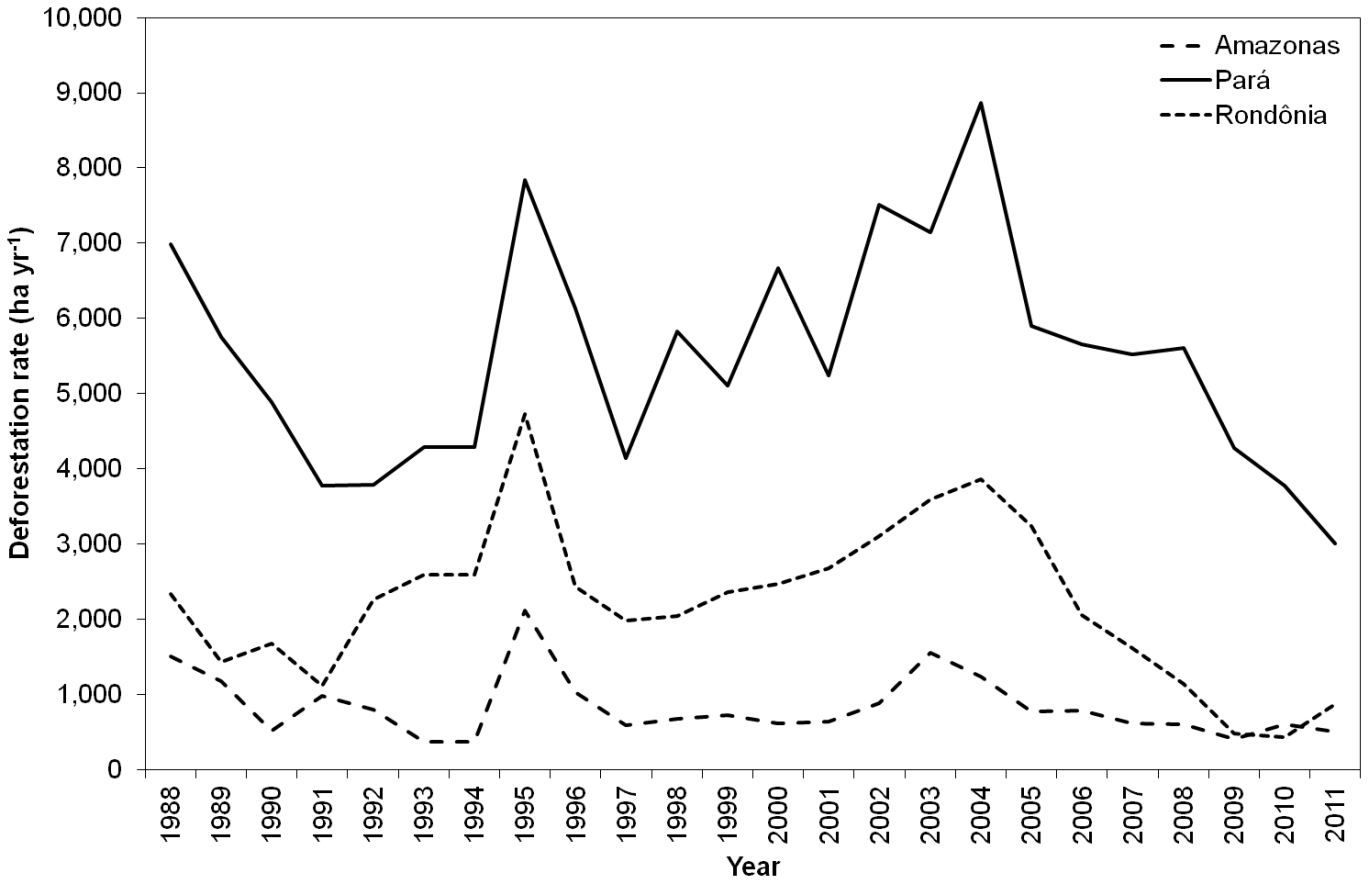
Deforestation rates in the Amazonas, Pará and Rondônia states according to INPE, 2013 [13]. Deforestation rates since 1988 (ha yr^−1^) in the Amazonas, Pará and Rondônia states according to the data reported by INPE, 2013 [13].

Extensive deforestation across the BLA started in the 1970s and was concentrated along the southern and eastern rims of the region [58]. The main deforestation drivers were conversion of forest to cropland and/or cattle ranching, which were carried out both by small farmers and large landholders [59]. According to the data provided by INPE, 2013 [13], the states where the three sites are located displayed different deforestation rates (Figure 10). Deforestation rates were always higher in Pará, followed by Rondônia and Amazonas. However, since 2003–2005, deforestation rates have been decreasing, with Rondônia experiencing a rapid decrease leading to values closer to those observed in the Amazonas state. Several factors contributed to this decline, namely the improvement of market-driven environmental governance [60]. Pará experienced the highest number of settled families by agrarian reform in the nine states composing the BLA (∼31,000 settled families per year in the period 2003–2006), and the number has been increasing since the 1960s [61]. Although the vast majority of deforestation in the BLA can be tracked to large landholders occupying the land for cattle ranching, the implementation of planned settlements is not negligible at all, especially in Rondônia, which is known by its small farmers’ radial, fishbone and watershed deforestation patterns (e.g., [58]). For example, up to the mid-1990s, the number of settled families in Rondônia was only second to Pará, with 1,423 settled families per year against 1,462 settled families per year in Pará [61].

The three study areas have different socio-economic drivers leading to deforestation, different land uses and subsequent regeneration. The Manaus site is mostly included in the Manaus municipality, although 24% is also included in the Rio Preto da Eva municipality. This study area basically encompasses several environmental protection areas. As such, it seems that these conservation areas have been critical at preserving most of the forest in the region. The conservation units (CUs) limits accessed from the Brazilian Ministry of the Environment (MMA, available at http://mapas.mma.gov.br/i3geo/datadownload.htm) were used to estimate the ratio of relative incidence of deforestation (NF and SF) outside and inside the CUs in the last date of the time-series for each site. The three sites had a significant proportion of the area included in the CUs: 43% in Manaus, 37% in Santarém and 23% in Machadinho d’Oeste. In Manaus, the relative incidence of deforestation outside the CUs was twice that inside CUs and in Santarém and Machadinho d’Oeste that value increased to five times. In fact, several studies have concluded that protected areas in the Amazon region are indeed effective at reducing deforestation rates (e.g., [60], [62], [63]). Figure 2A illustrates the lower amount of land cover change in Manaus, with approximately 20% of MF lost between 1973 and 2011, when compared with Santarém (more than 30% of MF lost between 1984 and 2010) and Machadinho d’Oeste (∼40% of MF lost between 1984 and 2011). Deforestation in the Manaus study area started in the 1970s, with the construction of the BR-174 highway connecting Manaus and Boa Vista. Early deforestation is traceable along this highway, but many areas were abandoned as a result of poor soil fertility; indeed, many small farmers moved to Roraima, which was considered to have younger fertile soils [64]. The area of forest in 2011 was approximately 69% and 81% of the territory of the Manaus and Rio Preto da Eva municipalities respectively [13]. Figure 2A shows that the proportion of MF in the Manaus site decreased from approximately 90% in 1973 to 71% in 2011, which is similar to the value reported by INPE, 2013 [13] for the Manaus municipality.

The Santarém site is located south of the city of Santarém, in the Belterra municipality, along the BR-163 highway, and including part of the Tapajós National Forest. This highway links Santarém and Cuiabá in the south. According to Scatena et al., 1996 [65] and Brondizio and Moran, 2012 [66], this region has a tradition of agriculture and agroforestry since the early 1900s, which has further escalated as a consequence of the construction of the BR-163 in the 1970s. In fact, of the three sites Santarém had the lowest proportion of MF at the beginning of the time-series (∼80%) and the highest proportion of SF (∼20%), thus suggesting that deforestation was commonplace in the area in the early 1980s. According to INPE, 2013 [13], approximately 68% of the forest area in the Belterra municipality was remaining in 2011, whereas we report a proportion of MF in 2010 of 46%. A vast majority of the Belterra municipality is included in the Tapajós National Forest, mainly the part that is west of the BR-163 highway. So, it is not surprising that the proportion of remaining forest in this municipality is higher than the proportion of remaining forest in the study area that includes the fraction east of the BR-163 highway that is not a protected area.

The Machadinho d’Oeste site (Rondônia state) is included mostly in the Machadinho d’Oeste municipality, although 20% overlaps the Vale do Anari municipality. Of the three sites, this was the one with the highest degree of land cover change, with a reduction of ∼60% in the MF cover from 1984 (91%) to 2011 (32%). Deforestation in this region started in the 1980s with the implementation of settlements by INCRA, of which Machadinho d’Oeste and Vale do Anari are just two examples [33]. According to Batistella and Moran, 2005 [33], the proportion of MF in 1988 and 1998 in Machadinho d’Oeste was around 80% and 66% respectively, while our estimates were 82% in 1987 (1988 was not mapped) and 55% in 1998. INPE, 2013 [13] reports an area of MF in the Machadinho d’Oeste municipality in 2011 of approximately 61%, and our study estimated the percentage of MF in the Machadinho d’Oeste study area as 32%. Google Earth data from 2013 shows that the areas of the Machadinho d’Oeste municipality to the east and north of the study area have much less deforestation and so this is a possible explanation for the higher proportion of MF detected by Batistella and Moran, 2005 [33] in 1998 and INPE, 2013 [13] up to 2011. Also, Batistella and Moran, 2005 [33] identified the land cover types occurring in the deforested areas of Machadinho d’Oeste. In 1988, approximately 2% was covered by SF, the value increasing to approximately 13% in 1998; our study mapped SF in 1987 and 1998 and the proportion was ∼13% and ∼29% respectively. Again, when considering the entire Machadinho d’Oeste municipality, there are extensive areas to the north and east of the study area that remained intact, with these covered by MF. Nevertheless, there is a coincident temporal trend that is the increase of SF from 1988 to 1998.

### 6.3. Land abandonment and the emergence of secondary forest

Land abandonment in the BLA is a consequence of a range of factors that have changed across the last decades since the inception of large scale deforestation in the region. Namely these are; reduced crop productivity as a consequence of poor soil fertility; lack of financial incentives, migratory patterns; non-traditional land uses and market fluctuations [67]. At Manaus, in 2011, land abandonment resulted in SF that was comprised mainly of areas with advanced ASF (50% ≥16 years), although large areas of intermediate ASF (37% between 6–15 years) were also present (Figure 3A and Figure 6). This suggests that the conservation areas were not only effective at preserving MF but also SF areas. As mentioned before, deforested areas in this study area were abandoned mainly because of poor soil fertility, with settlers moving to the nearby state of Roraima [64]. It is clear from Figure 3A that the higher proportion of advanced ASF in the study area is in the northern half of the site. These areas undergoing regeneration in 2011 had mostly short PALU (64%) as a consequence of intensification of land use and requirement for new land, and 65% was deforested only once. In comparison, the Santarém site, which included parts of the Tapajós National Forest, had regrowth dominated by intermediate ASF in 2010. Those areas experienced essentially short PALU (88% ≤2 years) and low FC (52% deforested once). According to Brondizio and Moran, 2012 [66], land abandonment in the region, and associated regeneration, was high until 1999. At this point, large-scale soybean cultivation started, mainly because of the decay of primary and secondary roads, lack of social services, and limited access to water. On the other hand, land abandonment in Machadinho d’Oeste resulted in SF that was mainly in the initial (46%) and intermediate (41%) ASF classes, with these areas experiencing mostly (75%) short PALU and low FC (57%). This was a planned settlement and most of the deforested area is under agriculture and or pasture use, suggesting that a vast majority of the area undergoing SF could be indeed forest fallow that might be under cattle ranching use or being subjected to subsistence agriculture in a crop/fallow cycle [66].

One important aspect worth mentioning is the large proportion of SF in the Santarém site that resulted from the 1997 wildfires. This area of regeneration was not the consequence of land abandonment but a natural process following fire-induced disturbance. This wildfire was just one among many others that occurred in the BLA as a consequence of the 1997–1998 *El Niño* Southern Oscillation (ENSO) event (e.g., [68]). In fact, the proportion of NF in 1997 (prior to the wildfire) was around 6% and in 1998 had increased to approximately 15%, mainly as a consequence of that large wildfire, perfectly visible in Figure 3B as the orange irregular zone of intermediate ASF in the centre of the study area. As a consequence of the natural regeneration process, the SF proportion increased to approximately 44% in 2000 (∼32% in 1997). Therefore, approximately 20% of the area of SF in 2010 was estimated as resulting from natural regeneration following that wildfire event.

### 6.4. Implications of prior land use for biomass accumulation and biodiversity restoration in secondary forests

Regenerating forests in the three selected sites have been classified in terms of ASF, PALU prior to abandonment and FC. Prior LULCC practices have a fundamental influence on the vegetation regenerating following land abandonment in the Amazon [10], [22], [69]–[75]. Vegetation structure, species composition and dominance are just some of the parameters of the regenerating vegetation that have been studied to identify the impact of prior land use practices (e.g., [76]). As such, these have an impact on the capability of these forests to accumulate biomass (and to act as a C sink at a greater or lesser extent) and to restore biodiversity (e.g., [77]).

Uhl et al., 1988 [71] studied areas undergoing secondary succession following pasture abandonment in Pará (Paragominas municipality) and identified major patterns related to prior land use intensity; light use sites, characterized by a lower PALU (0–4 years) and with no reclearance or just one slash-and-burn episode, had high species richness dominated by pioneer species (e.g., *Cecropia* sp. and *Solanum* sp.), and a ∼13 m closed canopy with an AGB accumulation rate of 10 Mg ha^−1^ yr^−1^ (8-year old sites); comparatively, moderate use sites, having higher PALU (6–12 years) and with 1 to 5 clearing episodes, had lower species richness dominated by a 7–8 m *Vismia* sp. partially developed canopy and an AGB accumulation rate of 5 Mg ha^−1^ yr^−1^; heavy use sites, subjected to large-scale mechanized operations, had poor species richness (*Solanum* sp. and *Cecropia* sp.), mainly composed by scattered trees and an AGB accumulation rate of only 0.6 Mg ha^−1^ yr^−1^. Mesquita et al., 2001 [75] studied regeneration pathways in a region north of Manaus that is included in our Manaus site (BDFFP) and identified major differences related to prior land use. Basically, areas that were deforested and abandoned immediately were dominated by *Cecropia* spp. trees, whereas those that were abandoned after some years of pasture use were dominated by *Vismia* spp. trees. Lucas et al., 2002 [22], also identified two distinct regeneration pathways in a region north of Manaus and in the same way as Mesquita et al. [75]; the authors identified *Vismia* spp. dominated regeneration associated to sites with more intensive land use and those dominated by *Cecropia* spp. to less intensive land use. According to Chazdon et al., 2007 [74], in terms of AGB accumulation, tropical secondary forests are characterized by a rapid growth rates in the first years after land (agriculture or pasture) abandonment. Furthermore, Zarin et al., 2001 [78] showed that there is a strong correlation between the AGB of secondary forests and the number of years following abandonment (i.e., ASF), soil texture and climate data in the Amazon region. On this pretext, the secondary forests in Manaus have the potential to accumulate more AGB, followed by Santarém and Machadinho d’Oeste. In 2011 (the last date of the time-series), Machadinho d’Oeste had approximately 45% of the area of SF with less than five years of age, and around 40% with less than three years of age. Machadinho d’Oeste was initially a settlement project implemented by the Brazilian government and today most of its people are dependent on subsistence agriculture [32]. Therefore, it is possible that some of the area mapped as SF was indeed forest fallow, part of a crop/fallow system of subsistence agriculture, which have been acknowledged by several studies (e.g., [79], [80]).

## Concluding Remarks

An accurate assessment of large-scale land use and land cover change dynamics over remote areas (e.g., Amazon) can only be carried out with an adequate monitoring system. Remote sensing data, in this case annual or quasi-annual time-series of high spatial resolution optical data (Landsat program), has proven its ability to accurately discriminate MF, SF and NF over three sites in the BLA experiencing several decades of deforestation. Lower deforestation rates and a greater proportion of intermediate and advanced ASF classes were characteristic of the Manaus site. On the other hand, Machadinho d’Oeste had the highest deforestation rates and lower regrowth rates, with most of its regeneration occurring in areas abandoned over the past 5 years. LULCC at Santarém displayed an intermediate behavior, with intermediate deforestation rates and the highest regrowth rates, which lead to regeneration dominated by areas of intermediate ASF. Conservation units were effective at reducing deforestation at all three sites. The temporal evolution of the spatial arrangement of the various parcels of land identified as one of the three classes could provide new insights about the fragmentation patterns in the region (Ewers et al. 2013 [81]).

Although several generalizations about the type and composition of secondary forest occurring in each of the three sites can be made, a correct assessment can only be made after an *in situ* assessment. Therefore, the research presented here will provide the opportunity to assess the influence of previous LULCC dynamics on the biomass accumulation and biodiversity restoration and to further investigate hypothesis related to differences among the three sites. A field campaign is foreseen for 2014 to collect forest inventory data that will be used to study the influence of prior LULCC on the capability of regrowth areas to accumulate AGB and to restore biodiversity. In the end, a framework related to the influence of prior LULCC on the biomass accumulation and biodiversity will be built and could be used to inform land management policies in the region.

## Supporting Information

File S1 **Relative and absolute deforestation and regeneration rates between consecutive dates for Manaus, Santarém and Machadinho d’Oeste.**
(DOC)
