# Supplementary material for: Land Use and Land Cover Change Dynamics across the Brazilian Amazon: Insights from Extensive Time-Series Analysis of Remote Sensing Data
Source: PLoS One. 2014 Aug 6;9(8):e104144. doi: 10.1371/journal.pone.0104144 (PMC4123946; doi:10.1371/journal.pone.0104144)
Supplement: File S1 — Relative and absolute deforestation and regeneration rates between consecutive dates for Manaus, Santarém and Machadinho d’Oeste. (DOC) [file pone.0104144.s001.doc]

**S1: Supporting Information**

**Relative and absolute deforestation and regeneration rates between consecutive dates for Manaus, Santarém and Machadinho d’Oeste**

Table S1. Deforestation and regrowth rates between consecutive dates in the Manaus time-series (MF - mature forest; SF - secondary forest).

| **Transitions (year)** | **MF annual rate of deforestation** | | **SF annual rate of deforestation** | | **Annual rate of deforestation** | | **Annual rate of regrowth** | |
| --- | --- | --- | --- | --- | --- | --- | --- | --- |
|  | **(% yr^-1^)** | **(ha yr^-1^)** | **(% yr^-1^)** | **(ha yr^-1^)** | **(% yr^-1^)** | **(ha yr^-1^)** | **(% yr^-1^)** | **(ha yr^-1)^** |
| [1973-1977] | 0.1 | 691 | 0.0 | 0 | 0.1 | 691 | 0.0 | 0 |
| [1977-1978] | 0.5 | 2,517 | 0.0 | 0 | 0.5 | 2,517 | 7.9 | 230 |
| [1978-1979] | 0.7 | 3,267 | 0.0 | 0 | 0.7 | 3,267 | 0.0 | 0 |
| [1979-1983] | 0.4 | 1,700 | 0.0 | 0 | 0.4 | 1,700 | 0.0 | 0 |
| [1983-1985] | 2.9 | 13,451 | 0.9 | 2 | 2.9 | 13,453 | 55.4 | 8,974 |
| [1985-1988] | 0.9 | 3,911 | 8.4 | 1,349 | 1.1 | 5,260 | 28.7 | 6,142 |
| [1988-1989] | 2.3 | 10,063 | 27.7 | 7,873 | 3.8 | 17,936 | 61.1 | 12,584 |
| [1989-1991] | 0.5 | 1,956 | 5.6 | 1,968 | 0.8 | 3,924 | 29.1 | 6,455 |
| [1991-1992] | 0.7 | 2,976 | 17.3 | 7,345 | 2.2 | 10,321 | 44.4 | 8,674 |
| [1992-1994] | 0.3 | 1,187 | 6.4 | 2,850 | 0.9 | 4,037 | 17.5 | 3,627 |
| [1994-1995] | 0.2 | 986 | 9.8 | 4,568 | 1.2 | 5,554 | 55.5 | 10,579 |
| [1995-1996] | 0.1 | 277 | 3.1 | 1,662 | 0.4 | 1,940 | 109.5 | 13,037 |
| [1996-1999] | 0.3 | 1,291 | 5.0 | 3,083 | 0.9 | 4,374 | 19.6 | 1,431 |
| [1999-2001] | 0.3 | 1,239 | 4.4 | 2,601 | 0.8 | 3,839 | 20.8 | 3,085 |
| [2001-2002] | 1.5 | 6,228 | 13.2 | 7,733 | 3.0 | 13,961 | 46.3 | 8,338 |
| [2002-2003] | 2.7 | 11,092 | 7.4 | 4,482 | 3.3 | 15,574 | 133.9 | 24,738 |
| [2003-2006] | 0.5 | 2,036 | 5.1 | 3,938 | 1.3 | 5,974 | 26.8 | 3,490 |
| [2006-2007] | 0.3 | 1,128 | 4.9 | 3,955 | 1.1 | 5,083 | 20.1 | 4,230 |
| [2007-2008] | 0.1 | 509 | 3.1 | 2,488 | 0.6 | 2,996 | 25.6 | 5,356 |
| [2008-2009] | 0.4 | 1,482 | 10.5 | 8,453 | 2.1 | 9,935 | 10.6 | 2,233 |
| [2009-2010] | 0.1 | 360 | 3.2 | 2,453 | 0.6 | 2,813 | 24.0 | 6,134 |
| [2010-2011] | 0.1 | 364 | 5.3 | 4,287 | 1.0 | 4,651 | 12.7 | 3,035 |
| Minimum | 0.1 | 277 | 0.0 | 0 | 0.1 | 691 | 0.0 | 0 |
| Maximum | 2.9 | 13,451 | 27.7 | 8,453 | 3.8 | 17,936 | 133.9 | 24,738 |
| Mean | 0.7 | 3,123 | 6.4 | 3,231 | 1.3 | 6,355 | 34.1 | 6,017 |
| Median | 0.4 | 1,591 | 5.0 | 2,725 | 0.9 | 4,513 | 24.8 | 4,793 |
| Inter-quartile range | 0.4 | 2,172 | 5.1 | 3,006 | 1.3 | 5,881 | 31.9 | 6,156 |
| Standard deviation | 0.8 | 3,726 | 6.5 | 2,699 | 1.1 | 4,905 | 33.7 | 5,756 |

Table S2. Deforestation and regrowth rates between consecutive dates in the Santarém time-series (MF - mature forest; SF - secondary forest).

| **Transitions (year)** | **MF annual rate**  **of deforestation** | | **SF annual rate**  **of deforestation** | | **Annual rate of deforestation** | | **Annual rate**  **of regrowth** | |
| --- | --- | --- | --- | --- | --- | --- | --- | --- |
|  | **(% yr^-1^)** | **(ha yr^-1^)** | **(% yr^-1^)** | **(ha yr^-1^)** | **(% yr^-1^)** | **(ha yr^-1^)** | **(% yr^-1^)** | **(ha yr^-1^)** |
| [1984-1985] | 1.5 | 1,467 | 28.9 | 2,883 | 4.1 | 4,350 | 83.9 | 1,708 |
| [1985-1986] | 2.2 | 2,075 | 30.1 | 2,683 | 4.6 | 4,759 | 79.0 | 3,190 |
| [1986-1987] | 1.8 | 1,674 | 3.6 | 386 | 2.0 | 2,060 | 182.9 | 6,332 |
| [1987-1988] | 3.2 | 2,964 | 82.3 | 9,401 | 12.0 | 12,366 | 40.9 | 897 |
| [1988-1989] | 0.45 | 420 | 6.6 | 531 | 1.0 | 952 | 136.4 | 10,350 |
| [1989-1990] | 0.9 | 796 | 17.2 | 2,856 | 3.5 | 3,651 | 126.0 | 3,330 |
| [1990-1991] | 0.9 | 826 | 20.3 | 3,404 | 4.0 | 4,230 | 116.2 | 3,362 |
| [1991-1993] | 4.5 | 3,817 | 7.4 | 1,272 | 5.0 | 5,089 | 60.7 | 2,200 |
| [1993-1995] | 0.9 | 710 | 10.8 | 1,983 | 2.8 | 2,694 | 79.5 | 4,808 |
| [1995-1996] | 1.4 | 1,100 | 23.6 | 5,475 | 6.5 | 6,575 | 32.9 | 2,070 |
| [1996-1997] | 1.5 | 1,175 | 6.2 | 1,349 | 2.5 | 2,524 | 84.8 | 7,016 |
| [1997-1998] | 15.6 | 11,046 | 23.4 | 5,906 | 17.7 | 16,952 | 261.5 | 7,063 |
| [1998-1999] | 2.5 | 1,617 | 48.7 | 11,352 | 14.7 | 12,969 | 113.3 | 11,726 |
| [1999-2000] | 2.0 | 1,286 | 11.0 | 3,104 | 4.8 | 4,390 | 102.3 | 12,257 |
| [2000-2001] | 0.4 | 235 | 6.0 | 2,285 | 2.5 | 2,520 | 63.6 | 4,920 |
| [2001-2003] | 0.9 | 531 | 9.8 | 3,710 | 4.3 | 4,241 | 11.1 | 832 |
| [2003-2005] | 2.2 | 1,301 | 10.6 | 3,434 | 5.1 | 4,735 | 20.0 | 2,760 |
| [2005-2006] | 0.8 | 459 | 3.2 | 1,079 | 1.7 | 1,538 | 18.8 | 3,238 |
| [2006-2007] | 0.9 | 493 | 1.3 | 482 | 1.0 | 975 | 45.9 | 6,385 |
| [2007-2008] | 0.9 | 499 | 12.6 | 5,036 | 5.7 | 5,535 | 10.5 | 1,183 |
| [2008-2009] | 0.4 | 254 | 0.5 | 194 | 0.5 | 448 | 27.4 | 3,861 |
| [2009-2010] | 0.7 | 404 | 3.2 | 1,354 | 1.8 | 1,758 | 30.3 | 3,359 |
| Minimum | 0.4 | 235 | 0.5 | 194 | 0.5 | 448 | 10.5 | 832 |
| Maximum | 15.6 | 11,046 | 82.3 | 11,352 | 17.7 | 16,952 | 261.5 | 12,257 |
| Mean | 2.1 | 1,598 | 16.7 | 3,189 | 4.9 | 4,787 | 78.5 | 4,675 |
| Median | 1.2 | 963 | 10.7 | 2,769 | 4.0 | 4,236 | 71.3 | 3,361 |
| Inter-quartile range | 1.3 | 1,085 | 16.6 | 2,350 | 3.0 | 2,831 | 79.6 | 4,031 |
| Standard deviation | 3.2 | 2,292 | 18.7 | 2,849 | 4.4 | 4,188 | 61.5 | 3,332 |

Table S3. Deforestation and regrowth rates between consecutive dates in the Machadinho d’Oeste time-series (MF - mature forest; SF - secondary forest).

| **Transitions (year)** | **MF annual rate of deforestation** | | **SF annual rate of deforestation** | | **Annual rate of deforestation** | | **Annual rate of regrowth** | |
| --- | --- | --- | --- | --- | --- | --- | --- | --- |
|  | **(% yr^-1^)** | **(ha yr^-1^)** | **(% yr^-1^)** | **(ha yr^-1^)** | **(% yr^-1^)** | **(ha yr^-1^)** | **(% yr^-1^)** | **(ha yr^-1^)** |
| [1984-1986] | 4.1 | 6,893 | 0.0 | 0 | 4.1 | 6,893 | 9.5 | 103 |
| [1986-1987] | 1.5 | 2,369 | 8.6 | 17 | 1.5 | 2,386 | 67.7 | 7,320 |
| [1987-1989] | 1.8 | 2,844 | 20.2 | 1,246 | 2.5 | 4,090 | 29.6 | 2,448 |
| [1989-1990] | 2.8 | 4,251 | 10.3 | 973 | 3.3 | 5,224 | 84.2 | 8,881 |
| [1990-1991] | 3.7 | 5,311 | 88.1 | 10,434 | 10.0 | 15,744 | 16.8 | 1,501 |
| [1991-1994] | 3.0 | 4,092 | 8.1 | 636 | 3.3 | 4,729 | 21.5 | 4,409 |
| [1994-1995] | 7.3 | 9,223 | 33.4 | 5,741 | 10.5 | 14,964 | 25.4 | 5,886 |
| [1995-1996] | 5.2 | 6,139 | 9.5 | 1,841 | 5.8 | 7,979 | 62.7 | 17,389 |
| [1996-1997] | 5.7 | 6,407 | 30.8 | 9,505 | 11.1 | 15,912 | 22.3 | 5,245 |
| [1997-1998] | 3.3 | 3,528 | 10.6 | 3,177 | 4.9 | 6,706 | 29.7 | 9,302 |
| [1998-1999] | 6.3 | 6,463 | 24.2 | 8,110 | 10.8 | 14,573 | 31.3 | 9,466 |
| [1999-2001] | 3.7 | 3,529 | 16.0 | 5,369 | 6.9 | 8,898 | 11.8 | 4,282 |
| [2001-2003] | 5.4 | 4,699 | 2.5 | 895 | 4.6 | 5,594 | 16.0 | 7,458 |
| [2003-2005] | 5.8 | 4,517 | 17.4 | 7,370 | 9.9 | 11,887 | 12.4 | 5,345 |
| [2005-2006] | 9.9 | 6,933 | 21.3 | 8,836 | 14.2 | 15,768 | 25.7 | 14,039 |
| [2006-2007] | 2.1 | 1,394 | 21.0 | 9,693 | 9.9 | 11,087 | 10.9 | 6,050 |
| [2007-2008] | 2.3 | 1,491 | 43.3 | 16,711 | 17.7 | 18,202 | 3.7 | 2,309 |
| [2008-2009] | 1.8 | 1,101 | 5.0 | 1,612 | 2.8 | 2,713 | 11.0 | 8,335 |
| [2009-2010] | 1.2 | 748 | 7.2 | 2,766 | 3.5 | 3,514 | 14.6 | 10,011 |
| [2010-2011] | 1.5 | 904 | 12.5 | 5,560 | 6.1 | 6,464 | 9.8 | 6,293 |
| Minimum | 1.2 | 748 | 0.0 | 17 | 1.5 | 2,386 | 3.7 | 103 |
| Maximum | 9.9 | 9,223 | 88.1 | 16,711 | 17.7 | 18,202 | 84.2 | 17,389 |
| Mean | 3.9 | 4,142 | 19.5 | 5,289 | 7.2 | 9,286 | 25.8 | 6,804 |
| Median | 3.5 | 4,171 | 14.3 | 4,273 | 6.0 | 7,436 | 19.1 | 6,172 |
| Inter-quartile range | 3.4 | 4,057 | 13.5 | 7,114 | 6.7 | 9,571 | 18.0 | 4,609 |
| Standard deviation | 2.3 | 2,394 | 19.5 | 4,467 | 4.4 | 5,248 | 21.5 | 4,141 |
